# O_2_ and Other High-Energy Molecules in Photosynthesis: Why Plants Need Two Photosystems

**DOI:** 10.3390/life11111191

**Published:** 2021-11-05

**Authors:** Klaus Schmidt-Rohr

**Affiliations:** Department of Chemistry, Brandeis University, Waltham, MA 02465, USA; srohr@brandeis.edu

**Keywords:** photosynthesis, bioenergetics, high-energy molecules, photosystems, charge separation, electron-transport chains

## Abstract

The energetics of photosynthesis in plants have been re-analyzed in a framework that represents the relatively high energy of O_2_ correctly. Starting with the photon energy exciting P680 and “loosening an electron”, the energy transfer and electron transport are represented in a comprehensive, self-explanatory sequence of redox energy transfer and release diagrams. The resulting expanded Z-scheme explicitly shows charge separation as well as important high-energy species such as O_2_, Tyr_Z_˙, and P680^+^˙, whose energies are not apparent in the classical Z-scheme of photosynthesis. Crucially, the energetics of the three important forms of P680 and of P700 are clarified. The relative free energies of oxidized and reduced species are shown explicitly in kJ/mol, not encrypted in volts. Of the chemical energy produced in photosynthesis, more is stored in O_2_ than in glucose. The expanded Z-scheme introduced here provides explanatory power lacking in the classical scheme. It shows that P680* is energetically boosted to P680^+^˙ by the favorable electron affinity of pheophytin and that Photosystem I (PSI) has insufficient energy to split H_2_O and produce O_2_ because P700* is too easily ionized. It also avoids the Z-scheme’s bewildering implication, according to the “electron waterfall” concept, that H_2_O gives off electrons that spontaneously flow to chlorophyll while releasing energy. The new analysis explains convincingly why plants need two different photosystems in tandem: (i) PSII mostly extracts hydrogen from H_2_O, producing PQH_2_ (plastoquinol), and generates the energetically expensive product O_2_; this step provides little energy directly to the plant; (ii) PSI produces chemical energy for the organism, by pumping protons against a concentration gradient and producing less reluctant hydrogen donors. It also documents that electron transport and energy transfer occur in opposite directions and do not involve redox voltages. The analysis makes it clear that the high-energy species in photosynthesis are unstable, electron-deficient species such as P680^+^˙ and Tyr_Z_˙, not putative high-energy electrons.

## 1. Introduction

Photosynthesis, the light-enabled synthesis of biomolecules from much simpler precursors, is a fascinating and obviously important process. For plants, the overall reaction is usually summarized as the production of glucose and oxygen,

6 H_2_O + 6 CO_2_ → C_6_H_12_O_6_ + 6 O_2_
Δ_r_*G*^o’^ = +2870 kJ/mol, Δ_r_*G*^o^ = +2875 kJ/mol = Δ_r_*G*^o”^. (1)

where a double prime indicates Alberty’s pH 7 biochemical standard conditions [1], a single prime conventional pH 7 biochemical standard conditions [2], and the absence of a prime chemical standard conditions (e.g., at pH 0); their differences are usually insignificant in this work. The solar energy is captured in a first step, the light reaction, which includes

12 H_2_O + 12 NADP^+^ → 12 NADPH + 12 H^+^ + 6 O_2_
Δ_r_*G*^o’^ = +2640 kJ/mol, Δ_r_*G*^o”^ = +2600 kJ/mol, (2)

as well as ATP production (“photophosphorylation”):

~18 (ADP + P_i_) → ~18 (ATP + H_2_O)  Δ_r_*G*^o’^ ≈ +540 kJ/mol. (3)

In the subsequent light-independent reaction, products of Equation (2), 12 NADPH + 12 H^+^, a biochemical equivalent of 12 H_2_ [2,3], go on to transfer hydrogen to 6 CO_2_ molecules to form carbohydrates like C_6_H_12_O_6_ (plus 6 H_2_O), with the needed moderate energy boosts provided by hydrolysis of the ATP molecules from Equation (3). Descriptions of the historical path towards the current understanding of photosynthesis can be found in excellent books [2,4] and articles [5,6,7,8].

Traditionally, the reactions in Equations (1) and (2) have been viewed as the production of energy-rich organic molecules, glucose and NADPH [2,9,10,11,12,13,14]. We have shown that the energetic aspect of this view is incorrect: The high-energy molecule among the products is O_2_ with its relatively weak double bond [15], while glucose and NADPH are only of moderate energy [3]. O_2_ shows its high energy in a myriad of strongly exothermic reactions with any of millions of organic molecules and many inorganic species, not only when forming CO_2_ and H_2_O, but also CO, formaldehyde, ethanol, acetic acid, glucose, SiO_2_, SO_2_, etc. [3,15]. The conclusion that O_2_ is a high-energy product making the reaction in Equation (2) unfavorable can be confirmed by considering an analogous reaction without O_2_ production:

12 H_2_ + 12 NADP^+^ → 12 NADPH + 12 H^+^
Δ_r_*G*^o’^ = −204 kJ/mol, Δ_r_*G*^o”^ = −252 kJ/mol, (4)

where free energy would be *released*.

In the most common representation of the energetics of oxygenic photosynthesis, the Z-scheme [2,4,12,13,16,17,18,19,20] (see Supplementary Material Figure S1), O_2_ often seems to be shown at very low energy [2,13,18], which cannot be correct [3,15]. Rectifying this problem was the original motivation for the present study. The resulting critical analysis also revealed that other high-energy molecules have been missing from the Z-scheme. In particular, the charged species in the important initial charge-separation process, emphasized in conceptual descriptions of photosynthesis [2,13,17,21], are absent from the Z-scheme. Furthermore, the labeling of the redox potentials in the Z-scheme inconsistently switches between reduced and oxidized species, or it shows conjugate redox species at different redox potentials (e.g., 2 H_2_O and O_2_ + 4 H^+^) [2,13,18]. The Z-scheme is easily misinterpreted as a diagram of energies of specific chemical species or electrons [4,20]. It incorrectly suggests that electrons spontaneously flow from H_2_O to ground-state chlorophyll, perplexingly releasing energy, before they are excited by photon energy. In this work, these problems (for a full listing see the SI) will be rectified, most importantly in a more comprehensive and intuitive diagram of relative free energies that contains the important high-energy species explicitly and is self-explanatory. In particular, it shows the energy boost by ionization of chlorophyll that enables Photosystem II (PSII) [22] to split water and produce O_2_, while the corresponding boost in Photosystem I (PSI) would be insufficient.

This analysis leads to a clear answer to the long-standing question why plants have two different photosystems working in tandem. Some textbooks just state this fact without explanation [2]. while others propose rationalizations that are unconvincing [12]. For instance, an ‘explanation’ that PSII is needed because it is hard to remove electrons from H_2_O [12] immediately requires the next explanation of *why* it is hard to remove electrons from H_2_O, e.g., compared to ubiquinol, QH_2_, the hydrogenated form of ubiquinone (coenzyme Q), which also features two O–H bonds that are broken when ubiquinol is oxidized. Experts declare [23] that “ancestor cyanobacteria had to evolve the capability to use two photosystems working in series in order to accumulate the energy of two photons”, which is an insufficient explanation since PSI already accumulates the energy of four photons, and since two photons in two Photosystems I in series, or two Photosystems II in series, could not achieve the needed net (i.e., formal) hydrogen transfer from H_2_O into NADPH. Our analysis reveals that photosynthesis in plants requires two distinct outcomes, each associated with a different photosystem: (i) PSII makes hydrogen from H_2_O available via plastoquinol for eventual bonding to CO_2_ and production of carbohydrates, (CH_2_O)_n_, and other biomolecules, while more of the photon energy ends up in O_2_ than in plastoquinol; and (ii) PSI converts solar energy into chemical energy in the organism, for instance stored first in H^+^ gradients and then in ATP; this occurs most prominently in “cyclic electron transport”, whose net effect is summarized by Equation (3) [2]. PSI also produces a less reluctant hydrogen donor, NADPH. As a “side effect” of the first process, the biosphere (rather than the specific organism) is supplied with chemical energy in the form of atmospheric O_2_, a high-energy molecule [3,15]. The opposing flows of energy and electrons [3] in photosynthesis and their implications for the misguided concept of “high-energy electrons” are discussed.

Before the outlined analysis, we provide a brief discussion of photosynthesis in the context of the laws of thermodynamics. Energy transport in antenna chlorophylls [4] is outside the scope of this paper.

## 2. Results

### 2.1. The Laws of Thermodynamics and Photosynthesis

The central energy boost in photosynthesis is sometimes treated implicitly as if the increase in the free energy of the plant is equal to the photon energy, Δ_r_*G* = *E*_photon_. However, this is not exactly what the laws of thermodynamics tell us, as outlined in the following paragraphs. Fortunately, the relative error is small.

The first law for the internal-energy change resulting from a process involving a closed system,

Δ*U* = *w* + *q* (5)

implies that energy is transferred either as work *w* or as heat *q* between the system and the surroundings. Work is usually generated by a force acting through a macroscopic distance. Heat is transferred through contact between bodies of different temperature, radiative heating, or friction (see Figure S2). Light energy from the sun is radiative heat [24]. This means that solar photon energy is heat and disproves a textbook statement [2] that heat is not a source of energy for cells.

The second law of thermodynamics states categorically that for a process occurring in a closed system at constant *T* and *P* [25],

Δ*G* < *w*_ele_. (6)

Since no external electrical energy is used to drive photosynthesis, *w*_ele_ ≤ 0 and the second law requires Δ*G* < 0 at constant *T* and *P*. How is it possible, then, that *G* increases in photosynthesis? The answer is found in the violation of the constant-*T* condition when heat is transferred from the sun at 5800 K to a leaf at 300 K. An analysis of photon emission and absorption [26,27] (see the SI, e.g., Equations (S37b) and (S42)) shows that the global entropy change in both emission and absorption is positive and allows for,

Δ_r_*G* < 0.92 *E*_photon_ > 0. (7)

This means that in the primary reaction in photosynthesis, Δ_r_*G* can be positive up to 0.92 *E*_photon_ ≈ +180 kJ/(mol photons). The factor of 0.92 in Equation (7) is slightly lower than the famous Carnot efficiency limit of (1−*T*_leaf_/*T*_sun_) = 0.95, as required: Like traditional heat engines, photosynthesis cannot fully convert heat (photon energy) to work [14,28,29,30,31] (note that Δ_r_*G* is equal to electrical work under suitable conditions) [32]. Nevertheless, due to the high temperature of the sun, the discrepancy between *E*_photon_ and the maximum Δ_r_*G* in Equation (7) is small and can usually be ignored. More specific models of photon absorption by chlorophyll [33] yield even lower, more stringent limits to the conversion of photon energy into free energy by the plant.

### 2.2. Misconceptions from Redox Potentials

Redox potentials (i.e., standard reduction potentials) are shown on the vertical axis in the Z-scheme and widely used in the quantitative description of photosynthesis. This section provides the link between redox potentials and the free-energy differences needed in our analysis as well as a critical discussion mostly intended for readers who have grown accustomed to redox potentials without being aware of their limitations. It explains why a different energy scheme of photosynthesis is desirable. Novices can ignore this historical detour and skip to Section 2.5. to learn the energetics of photosynthesis directly in terms of meaningful individual free energies *G*^o’^ of molecules and ions in kJ/mol, and their robust differences Δ_hr_*G*^o’^ in half reactions, as developed and shown in the figures below.

Redox potentials are given in volts, while energies in biochemical reactions should have units of kJ/mol. This use of inapplicable units obscures the understanding of bioenergetics. It conjures up an image of voltages that drive electric currents carried by high-energy electrons [18,34]; this is misleading, as shown in the following using the redox reaction “between Pheo and Q_A_”, i.e.,

Pheo^−^˙ + Q_A_ → Pheo + Q_A_^−^˙  Δ_r_*G*^o’^ = −35 kJ/mol (8)

as an example (see after Section 4. for short descriptions of Pheo and Q_A_; the raised dot indicates a radical). The redox potentials are *E*^o’^_Pheo, Pheo-·_ = −0.505 V and *E*^o’^_QA, QA-·_ = −0.144 V [35]:

- There is no voltage or electric potential difference [36] of [−0.144 V − (−0.505 V)] = 0.36 V between the locations of Pheo and Q_A_ in PSII, nor an associated electric field acting on electrons. The redox potential of *E*^o’^_Pheo, Pheo-·_ = −0.505 V depends on the free energies of *both* Pheo and Pheo^−^˙, and *E*^o’^_QA, QA-·_ = −0.144 V on those of both Q_A_ and Q_A_^−^˙ (see Equations (S63) and (S57)). Since Q_A_ and Q_A_^−^˙ are not simultaneously present in a given PSII, there is no physical basis for predicting a static voltage of 0.36 V. When Pheo^−^˙ sits at a distance *d* from Q_A_, the electric potential difference is roughly −*e/d*, which is unrelated to the redox-potential difference of 0.36 V. As in simple batteries, where positively charged ions move to the positive electrode [32], the movement of charged species in redox processes is not determined by electric fields or voltages but by free-energy (e.g., bond-energy) differences. An example of a redox reaction occurring without a voltage [37] is shown in Figure S5a and Equation (S95). The predicted voltage exists only if conductive electrodes, see Figures S5b–d and S6, are immersed into (or connected via salt bridges to) half cells each containing the reduced and conjugate oxidized species simultaneously in similar amounts.
- The energy release in a redox reaction such as Pheo^−^˙ + Q_A_ → … cannot be attributed to electron transfer from a high-energy donor to a lower-energy acceptor [3]. It is shown in the SI (see Equation (S52)) that the acceptor accepting the electron must be of high enough energy for the reaction to be spontaneous (Δ_r_*G*^o’^ < 0), since that acceptor (Q_A_ in our example) is a reactant.

A redox potential *E*^o^’ in volts can be formally converted to a free-energy difference in kJ/mol using the well-known relation,

Δ_hr,t_*G*^o’^ = −ν_e_ *F E*^o^’ (9)

with the stoichiometric coefficient ν_e_ of the electrons transferred in the half reaction (see the SI, e.g., Equation (S94)) and Faraday’s constant *F*. Here, the index “_hr_” refers to a half reaction, and the second subscript “_t_” to the traditional description (see the SI). When the Z-scheme is interpreted as an energy diagram [4,20], the free energy on the vertical axis is usually Δ_hr,t_*G*^o’^/ν_e_, at least implicitly. Redox potentials in V correspond directly to free-energy differences in eV.

Redox potentials are commonly shown as pseudo energy levels [18], prominently so in the Z-scheme [4,20], but the “redox-potential (free) energy” Δ_hr,t_*G*^o’^ obtained from a redox potential according to Equation (9) is the free energy neither of one specific chemical species nor of electrons:

- A redox potential depends on the bond and hydration energies of at least *two* chemical species [32], the oxidized and reduced molecules or ions in the half reaction, see Figure S3b, (as well as on the ionization energy for cations and the electron affinity of anions).
- Redox-potential energies are not energy levels of electrons. For instance, the electron energy in the electrodes of two separate electrochemical half cells connected only by a wire of negligible resistance is equal even when the half-cell redox potentials differ [3]. The energy of an electron in a half reaction depends on its environment and presents a difficult problem even in simple metal electrodes (keywords: work function, Fermi level, inner/outer/surface potentials) [38]; it would be even more challenging to analyze for electrons in molecules. ‘Free’ electrons are only intermediates and do not show up in the overall redox reaction such as Pheo^−^˙ + Q_A_ → Pheo + Q_A_^−^˙. Therefore, the energetics of batteries [32] and biochemical reactions [3] can be analyzed without requiring quantification of the unknown free energy of ‘free’ electrons.

What has nonetheless made redox potentials useful parameters is their formal relation to the free energy of reaction, Δ_r_*G*^o’^, see Equations (S92) and (S93). Thus, one can make quantitative predictions of Δ_r_*G*^o’^ from given empirical redox potentials, but they do not *explain* anything.

### 2.3. Why Electrons Move without Redox Voltages

The concept of redox voltages seems to provide an easy explanation for electron transport, but it is not valid. Why, then, do electrons move in an electron transport chain? The answer in the framework of classical thermodynamics refers to a reduction in Gibbs free energy: The electrons move so as to reduce the free energy by enabling a spontaneous chemical reaction with Δ_r_*G* < 0. Note that the reaction is not spontaneous because of the electron transfer, but because of the lower free energy of products relative to the reactants, due to stronger bonds, higher entropy, more favorable electron affinity for anions, lower ionization energy for cations, and/or more favorable hydration [32]. This is an energy-reduction argument that is quite simple and intuitive.

What thermodynamics really says is this: If electrons undergo net transport, it must be in the direction that ensures Δ*G* < *w*_ele_ = 0. This is a direct result of the second law of thermodynamics in the form of the inequality of Clausius evaluated at constant *T* and *P* [32]. This analysis correctly predicts that, referring to the reaction in Equation (8), the reactants may remain kinetically trapped for an indefinite amount of time, but if Pheo + Q_A_^−^˙ form by electron transfer, they will not undergo a net reverse reaction.

A somewhat more mechanistic and microscopic picture acknowledges that the electrons can move back and forth but will prefer to remain in relatively lower-free-energy Q_A_^−^˙ rather than higher-energy Pheo^−^˙, resulting in a net transformation of Pheo^−^˙ to Q_A_^−^˙. Correspondingly, Δ_r_*G*^o^ determines products of ratios of forward to reverse rate constants, *k*_f_/*k*_r_, even in an open system (see Equation (S110)). In the simplest case of an elementary reaction [39],

(10) $$\frac{k_{f}}{k_{r}}={\left(\frac{mol}{L}\right)}^{\Delta \nu }K={\left(\frac{mol}{L}\right)}^{\Delta \nu }\exp\left(-\frac{\Delta_{r}G^{o}}{RT}\right)$$

### 2.4. Misconceptions about Electron Donors

Good electron donors such as P680*, the excited state of the chlorophyll dimer at the center of Photosystem II, are mentioned prominently in textbook analyses of photosynthesis (for a brief description of this and other redox-active species in photosynthesis see the list after Section 4). It is easy to misunderstand what a statement like “P680* is an excellent electron donor” [2] really means. The half reaction in which an electron is donated is invariably energetically uphill (endergonic): No neutral stable chemical species reduces its free energy by giving off an electron, not even lithium metal, a strong reducing agent high up in a plot of −*E*^o^ with its very negative *E*^o^ = −3.04 V but still with Δ_hr_*G*^o^ = +119 kJ/mol > 0 for Li(s) → Li^+^(aq) + e^−^(g) [32]. Ionization of P700* to P700^+^˙ requires input of >250 kJ/mol, so P700* is not a spontaneous electron emitter either.

For a neutral species in photosynthesis to “act as an electron donor” means (i) ionization or (ii) loss of a hydrogen atom. Case (i), ionization, is equivalent to having a bound electron ripped off, which invariably requires energy input; as a simple example from introductory science, consider an isolated hydrogen atom in its ground state: for it to act as an electron donor, 13.6 eV = 1312 kJ/mol of energy has to be put in (an endothermic process). Case (ii), loss of a hydrogen atom from a molecule, means loss of the bond to the H-atom. Since bonds stabilize molecules and reduce their energy, loss of a bond by an electron donor means an energy increase; a simple example is presented in Figure S4. For instance, when an O–H bond in Tyr_Z_ is broken and the H-atom is given off (as a proton plus electron), high-energy Tyr_Z_˙ is formed. Even many singly negatively charged species (e.g., chloride or Pheo^−^˙ ions) give off their extra electron only if energy is put in (corresponding to the electron affinity of single atoms and the lowest unfilled molecular orbital (LUMO) binding energy of uncharged molecules).

“P680* is an excellent electron donor” means that P680* requires less energy input to give off its electron than many other electron donors; or equivalently, that when its electron is taken up by an average electron acceptor, the overall redox reaction reduces the system free energy and can be spontaneous. We could say colloquially that the electron in P680* is relatively ‘loose’.

### 2.5. The Self-Explanatory Expanded Z (EZ)-Scheme: Processes in Photosystem II (PSII)

**Figure 1:**
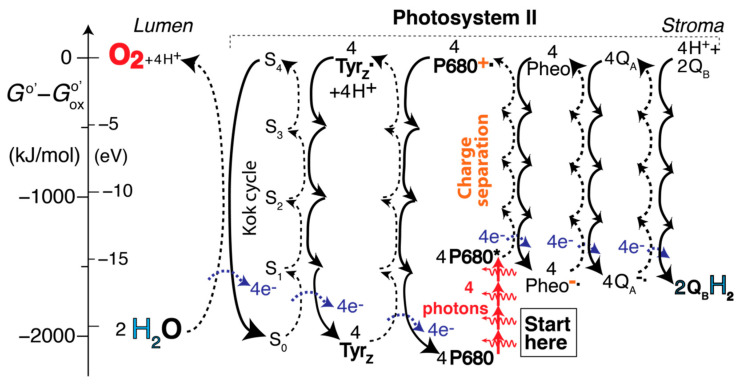
Series of redox-energy transfer and release diagrams of the series of light-initiated reactions in Photosystem II. Reductions releasing energy are indicated by solid downward curved arrows, oxidations by dashed upward curved arrows. On the vertical axis, 96.5 kJ/mol = 1 eV. The full names of the various abbreviated redox species are listed after Section 4 and in the SI.

The energy flow and chemical transformation in Photosystem II can be represented in a sequence of redox energy transfer and release (RETAR) diagrams [3] as shown in Figure 1. More energy is released in a reduction (downward arrow on a solid curved line) than is consumed in the oxidation (upward arrow on a dashed curved line) to which it is coupled in the redox reaction, which is therefore spontaneous. The relation between the free-energy change Δ_hr_*G*^o^’ associated with a curved arrow and the corresponding standard reduction potential *E*^o^’ in the traditional Z-scheme is,

Δ_hr_*G*^o^’ = −ν_e_ *F* (*E*^o^’+ 4.28 V), (11)

as derived in the SI (see Equation (S61)). An individual curved arrow is often associated with the transfer of one electron (ν_e_ = 1).

The first part of the expanded Z-scheme (EZ-scheme for short) of photosynthesis, shown in Figure 1, explains every step in PSII naturally: A photon excites P680, the chlorophyll dimer at the center of Photosystem II with an absorption maximum near 680 nm, to P680*, a state with a ‘loosened’ electron. (Four photons exciting four P680 are shown, because this is the number needed to generate one O_2_ molecule.) Pheophytin (“Pheo”) takes the loosened electron from P680*, turning it into P680^+^˙, which is high in energy for two reasons: because of the absorbed photon and because of the energy transferred from Pheo as it is reduced to Pheo^−^˙. The generation of P680^+^˙ and Pheo^−^˙ is charge separation, emphasized [2,13,17,21] because it prevents the undesirable conversion of P680* directly back to P680, which would just produce heat. Pheo taking up one electron is a fast process since it requires no co-reactant (unlike Tyr_Z_˙ also requiring H^+^ to form Tyr_Z_, or NADP^+^ requiring a second electron and a proton to form NADPH). Note that the reverse reaction of P680^+^˙ and Pheo^−^˙ would be energetically uphill and is therefore not spontaneous.

The diagram shows, to the left of center, how the high-energy electron-deficient P680^+^˙ rips an electron off of Tyr_Z_, turning it into Tyr_Z_˙ plus a proton. The high-energy, electron-deficient Tyr_Z_˙ in turn rips an electron off of H_2_O, mediated by the Mn_4_CaO_5_ water-splitting complex (also known as the oxygen-evolving complex or OEC), and combines with H^+^ back to Tyr_Z_. When this has happened four times (dissociating two H_2_O molecules) in a full Kok(-Joliot) cycle of the water-splitting complex, four H^+^ ions and one O_2_ molecule have been generated; [6] while the exact mechanism of the cycle is not currently known [8,21,40,41], as indicated in Figure 1 it must involve four (semi)stable intermediate states S_0_ to S_3_ and the unstable state S_4_, which reflect increasing oxidation states of Mn and deprotonated H_2_O generated using the energy transferred four times from P680^+^˙ [6]. The high-energy intermediates P680^+^˙, Tyr_Z_˙, and S_4_ are needed to generate the high-energy product, O_2_.

On the right side of the diagram, Pheo^−^˙ gets oxidized back to Pheo by Q_A_ spontaneously reducing the overall free energy and its oxidation state to Q_A_^−^˙ [35], which in turn gets oxidized back to Q_A_ by Q_B_ reducing the overall free energy and its oxidation state (towards Q_B_H_2_, in two steps). All of these redox processes are spontaneous because the free-energy reduction in the reduction half reaction is larger than the free-energy increase in the oxidation.

In the end, this process has effectively or formally moved four H atoms (or four protons and four electrons) from two H_2_O to two Q_B_H_2_, restored ground-state P680, and pumped four protons from the stroma to the lumen of the chloroplast:

2 H_2_O_lumen_ + (2 Q_B_ + 4 H^+^_stroma_) → (O_2_ + 4 H^+^_lumen_) + 2 Q_B_H_2_. (12)

The produced quinols (hydrogenated quinones) [2,35] are still much too low in free energy to transfer hydrogen to CO_2_ spontaneously:

12 Q_B_H_2_ + 6 CO_2_ → 12 Q_B_ + C_6_H_12_O_6_ + 6 H_2_O  Δ_r_*G*^o”^ = +1100 kJ/mol (13)

12 PQH_2_ + 6 CO_2_ → 12 PQ + C_6_H_12_O_6_ + 6 H_2_O  Δ_r_*G*^o”^ = +1260 kJ/mol (14)

where PQH_2_ is plastoquinol, the free form of Q_B_H_2_. The large positive values of Δ_r_*G*^o”^ in Equations (13) and (14) [35] make it clear why the further energy boost in Photosystem I is needed. The photon energy in PSII was mostly consumed to generate O_2_, a relatively weakly bonded, high-energy product [3,15]. The free-energy change in the water-splitting and O_2_-generating half reaction is easy to analyze using *G*^o’^ values of H_2_O(*l*), O_2_, and H^+^(aq) from Table S1 in ref. [3] (with stoichiometric coefficients of 2, 1, and 4, respectively):

Δ_hr_*G*^o^’^(ORR)^ = 2 × (−875.5) kJ/mol − (−463.5 kJ/mol + 4 × 170 kJ/mol) = −1968 kJ/mol. (15)

The sign here is opposite from that of the oxidation indicated by the left-most dashed curved arrow in Figure 1, since Δ_hr_*G*^o^’ by definition applies to the reduction reaction. It may be noted that this diagram does not *prove* that O_2_ is a high-energy molecule; other analyses [3,15] comparing the bonding in O_2_ with that in other molecules, without H^+^ involved, are needed to make this point convincingly. Nevertheless, O_2_ showing up high above H_2_O in the diagram is clearly *compatible* with O_2_ being energy-rich.

### 2.6. Processes Involving Cytochrome b_6_f, in the EZ-Scheme

Next, Q_B_H_2_ needs to be reoxidized to Q_B_ + 2 H^+^ for the water-splitting process in PSII to be able to occur again. After being released from PSII into the thylakoid membrane (quinones are relatively hydrophobic), Q_B_H_2_ is recognized as plastoquinol, PQH_2_, the protonated form of plastoquinone. PQH_2_ is central to a “Q-cycle” proton pump [2] associated with cytochrome *b*_6_*f*, whose function it is to move protons across the membrane from stroma (low [H^+^], pH = 8) to lumen (high [H^+^], pH = 5). The free energy put in to push the protons against a concentration gradient and electric potential is stored as chemical energy directly benefitting the organism; it is converted to chemical energy stored in ATP by the H^+^-flow-driven ATP synthase [2].

**Figure 2:**
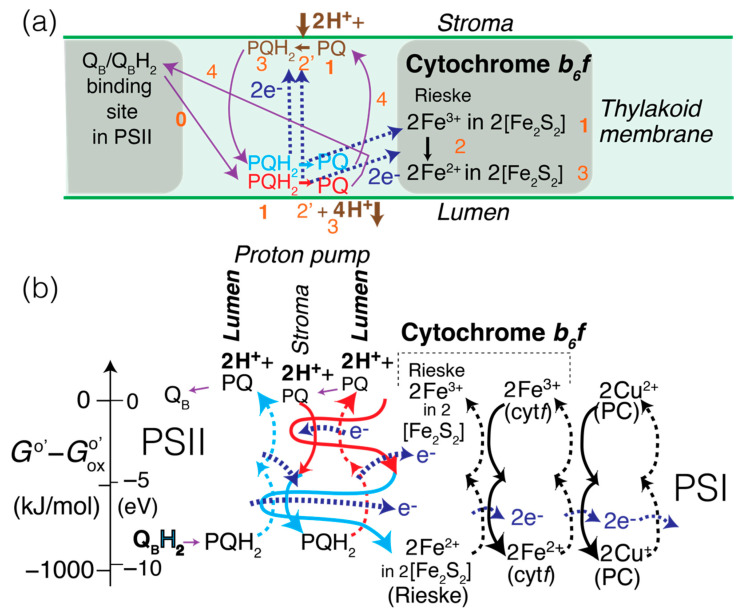
(**a**) Spatial schematic of the proton pump in the thylakoid membrane, which utilizes plastoquinol (PQH_2_) produced in PSII and (twice) the PSI-derived energy of an oxidized [Fe_2_S_2_] (or 2Fe-2S) iron–sulfur cluster in the Rieske protein of cytochrome *b_6_f* to pump protons from stroma to lumen. Numbering in orange indicates the sequence of steps in the overall process. (**b**) Series of redox-energy transfer and release diagrams associated with the proton pump and cytochrome *b_6_f* as well diffusible plastocyanin (PC), which couple the processes in PSII and PSI. For simplicity, the transfer of two electrons, rather than four as in Figure 1, is shown here. The full names of the various abbreviated redox species are listed after Section 4.

The Q-cycle is a complex process with three (rather than the normal two) coupled half reactions. In full versions of the Z-scheme, this complexity is reflected in unusual apparent energy increases (involving forms of cytochrome *b* subscripted “_6_” or “_L//H_”) rather than the usual downward trend [16]. A cartoon of the cycle (in spatial, not energetic terms) in the thylakoid membrane is shown in Figure 2a. The free-energy diagram of the Q-cycle in Figure 2b is color-coded to indicate that PQH_2_ undergoes two electron transfers (two red, stacked dashed curved upward arrows), one to Fe^3+^ in a [Fe_2_S_2_] (or 2Fe-2S) cluster of the Rieske protein, and the other to PQ at the other (stroma) side of the membrane. Concomitantly, PQH_2_ releases its H-atoms as protons into the lumen, while PQ picks up one proton from the stroma, forming PQH˙. To indicate that it is the substantial energy reduction of Fe^3+^ in [Fe_2_S_2_] that drives the whole process, its curved downward arrow in Figure 2b is expanded horizontally to cover the two other redox processes involved. Next, a second PQH_2_ (again at the lumen side) undergoes the analogous electron and proton releases, but in addition to a second reduction of Fe^3+^ in a [Fe_2_S_2_] cluster it now reduces the PQH˙ at the lumen side fully to PQH_2_ (again with a proton removed from the stroma). In short, the uphill proton-pumping redox reaction, with coupling through electron transfer via hemes at the center of the membrane,

(PQH_2_)_lumen side_ + PQ_stroma side_ + **2 H^+^_stroma_**

→ PQ_lumen side_ + **2 H^+^_lumen_** + (PQH_2_)_stroma side_ (16)

is driven by being coupled with energetically downhill

(PQH_2_)_lumen side_ + 2 [Fe^3+^_2_S_2_] (Rieske)
→ PQ_lumen side_ + **2 H^+^_lumen_** + 2 [Fe^2+^Fe^3+^S_2_] (Rieske). (17)

The reactions are coupled by (PQH_2_)_lumen side_ passing one electron to Fe^3+^ in [Fe_2_S_2_] and one to PQ_stroma side_ + 2 H^+^_stroma_, so more realistically each of the two (PQH_2_)_lumen side_ contributes half to the first and half to the second reaction shown. All PQH_2_ are at the same energy, so the slightly higher energy of the protons in the lumen relative to the stroma shows at the top of the diagram in Figure 2b.

The electron transport from left to right and free-energy flow from right to left through cytochrome *b*_6_*f* continues in Figure 2b with regular redox pairs: The reduced iron–sulfur cluster in a Rieske protein is oxidized by Fe^3+^ in cytochrome *b*_6_*f* reducing to Fe^2+^, which in turn is reoxidized by Cu^2+^ in plastocyanin reducing to Cu^+^.

### 2.7. Processes Involving Photosystem I (PSI) in the EZ-Scheme

**Figure 3:**
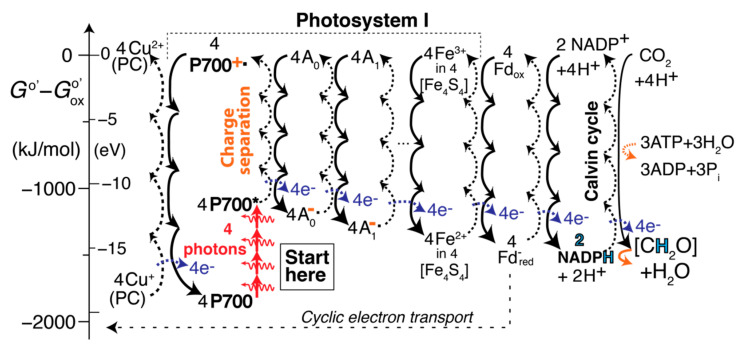
Series of redox-energy transfer and release diagrams associated with Photosystem I. The full names of the various abbreviated redox species are listed after Section 4. The energy contribution of 3 ATP in the Calvin cycle is shown by a curved orange arrow (shown separately as well as adding to the other downward arrow in the lower right corner).

Plastocyanin is water-soluble and diffuses to PSI, where its copper center is oxidized back to Cu^2+^ with energy provided by P700^+^˙ when the process continues as shown in Figure 3. Photon energy absorbed by P700 (a chlorophyll dimer with an absorption maximum near 700 nm) loosens an electron, forming excited P700*, which is easily ionized and raised in energy to P700^+^˙ by chlorophyll A_0_, which gives off energy by taking up the loosened electron and forming A_0_^−^˙. A_0_ is quickly restored by reduction of A_1_. Next in the diagram, three dots indicate two regular redox steps that have been skipped for brevity because their redox energy cycles are analogous to others shown. These involve two [Fe_4_S_4_] or 4Fe-4S clusters that cycle between Fe^3+^ and Fe^2+^, just like the third cluster, which is shown in the figure. Fe^2+^ in [Fe_4_S_4_] is cycled back to Fe^3+^ by reduction of Fe^3+^ in an [Fe_2_S_2_] cluster in ferredoxin, a soluble protein. Reduced ferredoxin can initiate cyclic electron transport, which is discussed further below, or be oxidized back to Fe^3+^ by redox reaction with NADP^+^, which is catalyzed by ferredoxin–NADP^+^ reductase (FNR) [2].

### 2.8. “Hydrogenation” (Reduction) of CO_2_ to Carbohydrates

The EZ-scheme concludes in Figure 3, on the right, with the restoration of NADP^+^ and carbohydrate [CH_2_O] synthesis by transfer of hydrogen to CO_2_, in the Calvin cycle (greatly simplified). The curved orange arrows indicate that ATP hydrolysis is needed to make the redox reaction spontaneous. The surprising status of CO_2_ among the higher-energy species here is explained in terms of bond energies in the SI. In short, the reduced species, [CH_2_O] + H_2_O, have two more electron-pair bonds than CO_2_ + 4 H^+^(aq) and therefore appear lower in energy; the ionization energy of a hydrogen atom in water at pH 7, +170 kJ/mol (see Figure S4b) [3], also raises the energy of the reduced species. (In terms of energy per electron-pair bond, which is generally a more meaningful measure of the energetics of molecules [15], CO_2_ and [CH_2_O] are actually very similar, see also the SI, Equation (S113).) Hypothetical energy-releasing reactions of CO_2_ + 4 H^+^ with various reduced species in PSI, see Equations (S114) and (S115), confirm the inferred energetic status of CO_2_ + 4 H^+^.

### 2.9. Cyclic Electron Transport

In cyclic electron transport, PSI runs the Q-cycle proton pump without O_2_ production. This is shown in Figure S9. The details of how ferredoxin is reduced by a quinone needed in the Q-cycle are possibly not fully understood. This process exclusively produces chemical energy directly for the plant, stored in a proton concentration gradient that is utilized for ATP synthesis. It is thus unlike O_2_ production in PSII, which does not directly benefit the plant, and unlike true photo-*synthesis* producing carbohydrates by transfer of hydrogen to CO_2_.

### 2.10. The Complete EZ-Scheme of Oxygenic Photosynthesis

**Figure 4:**
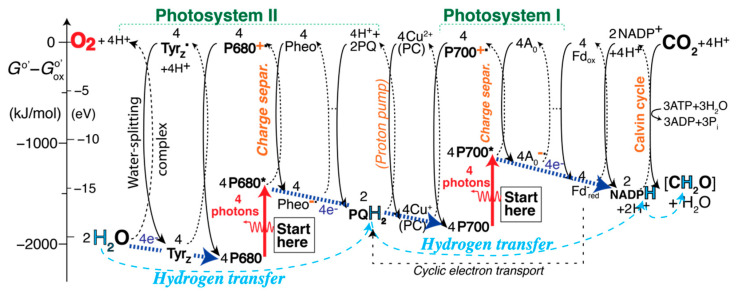
Simplified comprehensive EZ-scheme of photosynthesis in plants, converting H_2_O and CO_2_ (lower left and upper right corners, respectively) to O_2_ and carbohydrate [CH_2_O] (remaining corners). The broad straight dark-blue arrows indicate transfer of four electrons between electron-rich species, tracing out the familiar Z-pattern. The thin curved light-blue arrows at the bottom highlight net or formal hydrogen transfer. A more detailed version of the scheme is shown in Figure S10.

Figure 1, Figure 2 and Figure 3 show the EZ-scheme broken up into three pieces for clarity. The full sequence of reactions is displayed in Figure S10. A simplified, more compact version is shown in Figure 4, with charge separation in the photosystems, generation of high-energy oxygen, and the Calvin cycle still included; only the Kok cycle and the complicated proton pump are not shown in an intelligible manner. Straight blue arrows at the bottom trace out the familiar Z-scheme of the reduced species, which is, however, greatly expanded by inclusion of the energetics of the high-energy oxidized species shown at the top.

### 2.11. Vertical Shifts in the EZ-Scheme

In a redox energy transfer and release diagram, the “column” for a given half reaction can be shifted up or down without changing the energy released in reduction and the energy used in oxidation. For instance, while the free energies of P680, P680*, and P680^+^˙ are fixed relative to each other, they are not in a fixed relation to the free energy of Pheo, which, however, is fixed relative to Pheo^−^˙.

When comparing the bond energies of molecules [15] and ionization energies in water [32], the free atoms are a natural reference point. They were chosen as the zero point of the energies in Figure S11, which shows the higher energy of P680^+^˙ compared to P700^+^˙ more clearly but looks more disjointed than Figure 4. For many species, the thermodynamic data needed for Figure S11 are not easily available. If we set the free energies of all the oxidized species to zero as in Figure 4, a relatively smooth diagram results in which the reduced species trace out the familiar Z-scheme.

### 2.12. An Alternative EZ-Scheme: Energy Flow

**Figure 5:**
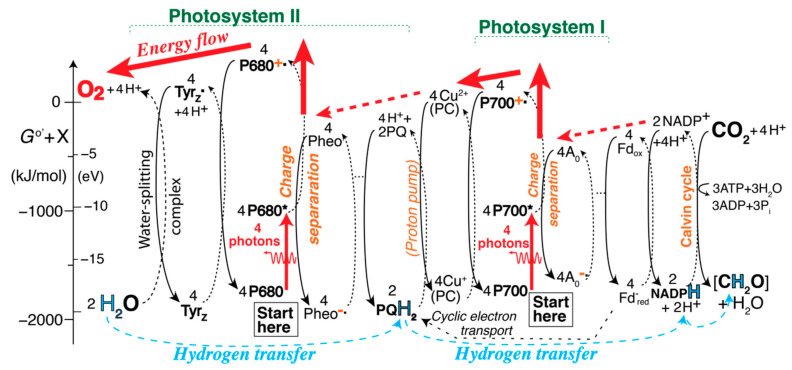
Alternative simplified EZ-scheme of photosynthesis in plants, converting H_2_O and CO_2_ (lower left and upper right corners, respectively) to O_2_ and carbohydrate [CH_2_O]. The vertical shift X of each half-reaction “column” was adjusted to facilitate energetics comparisons, e.g., between P680^+^˙ and P700^+^˙. The direction of energy transfer and release is indicated by straight red arrows at the top, formal hydrogen transfer by dashed curved arrows at the bottom of the diagram. Three dots … indicate omitted redox reactions. A more detailed version of this scheme is shown in Figure S12.

An alternative version of the comprehensive EZ-scheme is presented in Figure 5 and Figure S12. It again takes advantage of the mentioned free choice of the vertical displacement of each half-reaction column in order to make various energetics comparisons easier. Neutral reduced molecules are shown guided by bond-energy diagrams [3,15], as in Figure S11, while ionic reduced species are aligned with their right neighbors for a simpler diagram. With ground-state P680 and P700 assigned the same energy, the easier ionization of P700* to P700^+^˙, compared to P680* to P680^+^˙ [42], becomes more apparent than in Figure 4. The relative energies of the “active bonds” in the hydrogen carriers H_2_O, Tyr_Z_, Q_B_H_2_/PQH_2_, NADPH + H^+^, and [CH_2_O] + H_2_O match bond-energy considerations outlined in Section 3.7. below. The red arrows at the top indicate energy flow from PSII to the water-splitting complex and into high-energy O_2_, and from PSI to the proton pump (and thus, eventually, to ATP). Ancillary energy flow is marked by thinner dashed red arrows; for instance, the energy boost of P680* to P680^+^˙ comes from the right. While energy transfer is not the main function of photosynthesis, the crucial net or formal hydrogen transfer from H_2_O to carbohydrate marked at the bottom of the diagram can occur only if the intervening redox reactions, enabled by photon absorption, release free energy.

### 2.13. The Corrected Z-Scheme

**Figure 6:**
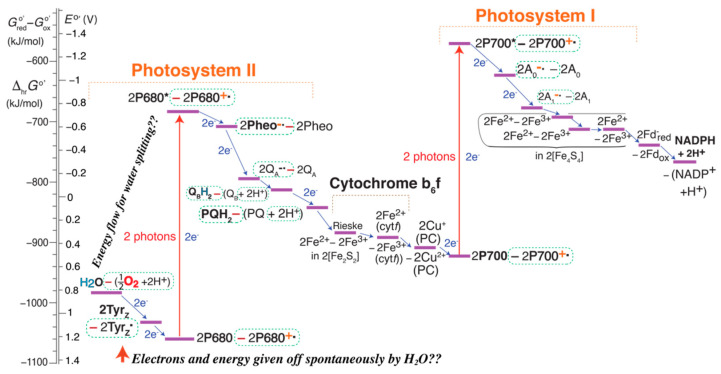
Corrected Z-scheme. Each “energy level” represents the difference Δ_hr_*G*^o^’ in energy between the reduced and oxidized species in the half reaction, as shown quantitatively on the left vertical axis, with stoichiometric coefficients as shown (consistently with transfer of two electrons). The difference between the reduced and oxidized species indicated at a given “energy level” refers to their difference in standard free energy *G*^o’^. The quantities on the two vertical axes are linearly related, Δ_hr_*G*^o^’ = −*F* (*E*^o^’+ 4.28 V). Important species missing from the uncorrected traditional Z-scheme (some oxidized, others reduced) are marked by dashed green boxes.

The EZ-scheme helps clarify the original Z-scheme and makes it clear how the Z-scheme can be made more consistent and meaningful. Each pseudo-energy-level in the Z-scheme indicates the free-energy *difference* between products and reactants in a reduction half reaction (see ref. [3] and Figure S3b), i.e., between reduced and oxidized species. This is indicated in Figure 6; for brevity, the symbol *G*^o^ has been omitted for all species. For instance, “H_2_O—(½ O_2_ + 2 H^+^)” means

*G*^o^_H_2___O_—(½ *G*^o^_O_2__ + 2 *G*^o’^_H^+^_). (18)

While some references have shown both oxidized and reduced species in the Z-scheme, they usually left out protons, even though these are crucial electron acceptors [3], and used the division slash instead of the minus sign between reduced and oxidized species [35]. Previously missing species such as O_2_, Tyr_Z_˙, P680^+^˙, P700^+^˙, and Pheo^−^˙ all show up in the corrected Z-scheme in Figure 6. Nevertheless, the high energies of the former three are still not directly obvious, unlike in the EZ-schemes of Figure 4 and Figure 5.

## 3. Discussion

### 3.1. The Superiority of the EZ-Scheme

The EZ-scheme shown in Figure 1, Figure 2, Figure 3, Figure 4 and Figure 5 and Figure S12 eliminates the mentioned shortcomings of the Z-scheme: It displays charge separation and the two sources of the high energy of P680^+^˙ (the absorbed photon and Pheo) explicitly; it shows O_2_ correctly at high energy; it explicitly shows both reduced and oxidized species; it demonstrates that PSI has insufficient energy to split water and produce O_2_ because P700* is easier to ionize than P680* [42], which also clarifies why P700 is shifted up relative to P680 in the traditional Z-scheme and why two photosystems are needed; it shows the energy flow from P680^+^˙ to H_2_O needed for water splitting and explains that electrons move from H_2_O to Tyr_Z_˙ because the coupled reduction of the latter and oxidation of the former has Δ*G* < 0; it makes clear that the process starts with the absorption of a photon; it shows that 2 × 4 photons are needed per O_2_ produced; it can represent pure energy transfer (see Figures S7 and S8); the “energy levels” represent the (relative) energies of chemical species [3] rather than energy *differences* that are easily misinterpreted; it explains the Q-cycle proton pump and includes the Kok and Calvin cycles.

### 3.2. PSI Has Too Little Energy for Water Splitting, Due to Low Ionization Energy

The physical difference between PSII and PSI is not apparent from the traditional Z-scheme, where PSI is shifted up, without an intuitively meaningful explanation. The EZ-scheme, by contrast, makes clear why: the upshift in *E*^o’^ corresponds to a smaller Δ_hr_*G*^o’^, which is due to a smaller energy difference between P700^+^˙ and P700, compared to P680^+^˙ and P680. This difference between the energy differences is directly apparent in the EZ-scheme of Figure 5 and Figure S12. The energy of P700^+^˙ relative to ground-state P700 is the sum of the absorbed photon energy and the ionization energy of P700* (to P700^+^˙), and an analogous statement applies to P680^+^˙. Since the absorbed-photon energies differ only by about (700−680)/700 = 3% or ~10 kJ/mol, the main energetic difference between P680* and P700* is the ~80-kJ/mol smaller ionization energy of the latter, which has been attributed to differences in the protein atomic charges [42]. As a result of this reduced ionization energy, the ionized form P700^+^˙ has too little energy to split water and generate O_2_. In a traditional *E*^o’^ diagram, this is indicated by an *E*^o’^ value < 0.82 V for P700–P700^+^˙.

This analysis shows that a textbook statement [2] to the effect that P680* is an excellent electron donor misses an important point and needs to be qualified. P700*, being more easily ionized, is actually a more excellent electron donor, but it must be pointed out that this is not always an advantage. For instance, it is the reason why P700* after donating an electron has too little energy to split water and produce O_2_.

### 3.3. Little of the Energy of the Photons Absorbed by PSII Directly Benefits the Plant

We argue that most of the energy of the photons absorbed by PSII is used to produce high-energy dioxygen, which leaves the leaf and therefore does not benefit the plant directly. What the water-splitting PSII produces within the plant is plastoquinol, PQH_2_, which is a rather low-energy molecule, in part due to aromatic stabilization. Consider that the reaction of 12 PQH_2_ with 6 CO_2_ to glucose and water, Equation (14), would be strongly uphill energetically, by Δ_r_*G*^o”^ = +1260 kJ/mol. We can compare this with much less unfavorable hydrogenation of CO_2_ from H_2_

12 H_2_ + 6 CO_2_ → C_6_H_12_O_6_ + 6 H_2_O
Δ_r_*G*^o^ = +31 kJ/mol, Δ_r_*G*^o”^ = +27 kJ/mol (19)

and with the spontaneous, energy-releasing reaction that CO_2_ could undergo with the products of PSI, i.e., NADPH and ATP:

12 NADPH + 12H^+^ + 12 ATP + 6 CO_2_ → 12 NADP^+^ + C_6_H_12_O_6_ + 12 ADP + 12 P_i_
Δ_r_*G*^o’^ < −125 kJ/mol, Δ_r_*G*^o”^ < −90 kJ/mol. (20)

This demonstrates that PSI provides significant energy for the organism, while PSII does not. A literature claim ([2], p. 779) that “the percentage of the photon’s energy conserved in PQH_2_ … is 30%” incorrectly attributes the energy of O_2_ [3,15] to PQH_2_. This energy is not intrinsic to PQH_2_ and would not be available in an anaerobic environment, while the energy of O_2_ could be released by reaction with any organic molecule, H_2_, H_2_S, C(s), P_4_(s), S_8_(s), or Fe(s). That PQH_2_ is a relatively low energy species is confirmed by the observation that it has no energy-releasing reaction with any organic biomolecule:

12 PQH_2_ + 12 (CH_3_)_2_C=O → 12 PQ + 12 (CH_3_)_2_CHOH
Δ_r_*G*^o”^ = +922 kJ/mol (21)

12 PQH_2_ + 12 pyruvate → 12 PQ + 12 lactate
Δ_r_*G*^o”^ = +695 kJ/mol (22)

12 PQH_2_ + 12 oxaloacetate → 12 PQ + 12 malate
Δ_r_*G*^o”^ = +635 kJ/mol (23)

12 PQH_2_ → 12 PQ + 12 H_2_  Δ_r_*G*^o”^ = +1230 kJ/mol (24)

12 PQH_2_ + 12 fumarate → 12 PQ + 12 succinate
Δ_r_*G*^o”^ = +200 kJ/mol (25)

Even a C=C double bond as a fairly high-energy co-reactant in Equation (25) is not sufficient to make the reaction spontaneous. Only biochemical reactions of PQH_2_ with certain Fe- or Cu-containing species or with O_2_ are spontaneous.

### 3.4. Two Photosystems Because of the High Energy of O_2_

The function of processes in Photosystem II is not conversion of solar to chemical energy, but setting the stage for the *synthesis of organic molecules*: taking H-atoms (as H^+^ + e^−^) from H_2_O in order to (formally) transfer them to CO_2_ and eventually produce glucose and other biomolecules. This has been appreciated by some authors (though others see the function differently [4], e.g., in energizing electrons [43]). What has rarely been recognized is that the photon energy in Photosystem II is essentially used up to make the high-energy molecule O_2_, a “waste product” that does not directly benefit the organism. Therefore oxygen-producing organisms need a second photosystem, PSI, to generate chemical energy for the organism, for instance in the form of ATP.

### 3.5. Photosynthesis with Only One Photosystem

As a test of our understanding of the need for two photosystems in oxygenic photosynthesis, it is instructive to compare with anoxygenic microbial photosynthesis. As predicted by our analysis, without the high energetic cost of O_2_ production, a single photosystem is sufficient here.

Photoorganoheterotrophs (such as the heliobacteria or some filamentous anoxygenic phototrophs, also known as green non-sulfur bacteria [4,12,17]) utilize organic C and H (e.g., of acetate, succinate, or pyruvate) rather than CO_2_ and H_2_O as their carbon and hydrogen sources. These organisms only need to convert given organic molecules into carbohydrates, which requires relatively little energy, e.g.,

3 CH_3_COOH → C_6_H_12_O_6_   Δ_r_*G*^o^ = +271 kJ/mol. (26)

This reaction is 10 times less endergonic than the glucose production in plants according to Equation (1). The single microbial photosystem can use photon energy to produce ATP that can make slightly endergonic reactions like Equation (26) spontaneous, analogous to the reaction between NADPH + H^+^ and CO_2_ in Equation (20), which would be slightly endergonic without ATP hydrolysis.

Some photolithoautotrophs use H_2_ (e.g., purple non-sulfur bacteria in photoautotrophic mode) or H_2_S (purple or green sulfur bacteria) [4,17] as their source of hydrogen to produce glucose. According to Equation (19), synthesis of glucose from H_2_ and CO_2_ without O_2_ production is only slightly exergonic, Δ_r_*G*^o^ = +31 kJ/mol, and from H_2_S and CO_2_ it only requires a moderate amount of energy input,

6 CO_2_ + 12 H_2_S → C_6_H_12_O_6_ + 6 H_2_O + 1.5 S_8_
Δ_r_*G*^o^ = +426 kJ/mol. (27)

Because of the relatively weak bonds in H_2_S compared to H_2_O and because these reactions do not produce high-energy O_2_, a single photosystem is sufficient to supply the few hundred kilojoules that are needed per mole of glucose produced.

### 3.6. Photon Energy Stored in O_2_ vs. Glucose

The net result of photosynthesis is mostly the synthesis of glucose and oxygen according to Equation (1). It had long been assumed that the required input of +2875 kJ/mol is chemical energy stored in the bonds of glucose [2,14]. We have shown that this is incorrect: due to its weak double bond, O_2_ is a high-energy molecule, while glucose is only of moderate energy [3,15]. This can be confirmed by comparing the total bond-formation energies (enthalpies) of the four species involved:

6 H_2_O(*l*) + 6 CO_2_ vs. C_6_H_12_O_6_
**+ 6 O_2_** (28)

Bond-formation energies:

6(−971) + 6(−1608) vs. −9672 + 6 (−498) kJ/mol (29)

−5826 + (−9648) vs. −9672 + (**−2988**) kJ/mol. (30)

These data show that the oxygen molecules are the least negative, i.e., highest, in energy among the four different species.

The analysis just presented tends to assign an only moderately negative (i.e., a relatively high) energy to molecules with few bonds or atoms. A more meaningful comparison can be made in terms of the average energy of two electron-pair bonds (in kJ/mol) in the molecules because the number of electron pair bonds is the same in reactants and products (if O_2_ is assigned its usual bond order of two); then a reaction will occur spontaneously from reactants with weak bonds to products with stronger bonds [15]. The following data comparing the energies of pairs of electron-pair bonds

H_2_O(*l*)  CO_2_ vs. C_6_H_12_O_6_  **O_2_**
2 electron-pair bonds: −971 −804  −806 **−498** kJ/mol (31)

confirm that O_2_ is the highest-energy species in the reaction. It is interesting to note in Equations (30) and (31) that the total energy of the 24 electron-pair bonds in 6 CO_2_ and C_6_H_12_O_6_ is essentially the same.

The average energy per electron-pair bond in the reactants of Equation (31) is −860 kJ/mol. The energy of glucose is by 24/2 × (860 − 804) = 672 kJ/mol, that of 6 O_2_ by 6 × (860 − 498) = 2172 kJ/mol higher than that of the reactants. Thus, 76% of the energy of the products can be assigned to O_2_, as usual [15]. The corresponding analysis in terms of free energies, with similar results, is shown in the SI (Equation (S116)). Overall, more than 3/4 of the chemical energy produced in photosynthesis is stored in O_2_ rather than glucose.

### 3.7. Energetics of H_2_O Relative to O_2_ and QH_2_

The photosynthesis reaction of Equation (1) is energetically uphill because of the relatively strong bonds in the reactant H_2_O and the relatively weak double bond of the product O_2_, while according to Equation (30), the 24 electron-pair bonds in 6 CO_2_ and glucose essentially balance each other out energetically. The energy contributions of H_2_O and O_2_ can be reasonably separated by considering the energetics of Equation (1) if it produced a hypothetical O_2_′ molecule with a more typical pair-of-electron-pair bond strength of −806 kJ/mol, which would be within ±2 kJ/mol (i.e., within ± 0.3%) of ½ CO_2_ and the average for glucose (see Equation (31)) and still weaker than in ½ CH_4_ (−815 kJ/mol) and 2 H_2_ (−872 kJ/mol). With oxygen’s experimentally observed entropic free-energy contribution of 498 − 464 = 34 kJ/mol, the free energy of the hypothetical O_2_′ is −772 kJ/mol, 308 kJ/mol below the actual −464 kJ/mol of O_2_. Accordingly, O_2_ not being weakly bonded would make the photosynthesis reaction of Equation (1) less unfavorable by 6 × (−308 kJ/mol) = −1848 kJ/mol. The remaining free-energy difference of 2875 − 1848 = 1027 kJ/mol can be attributed to the relatively strong bonds in 6 H_2_O compared to glucose, CO_2_, and O_2_′. This value of 1027 kJ/mol is similar to the 1260 kJ/mol required for glucose synthesis from PQH_2_ and CO_2_ according to Equation (14) and justifies placing 2 H_2_O and 2 PQH_2_ at approximately the same energy in Figure 5. This confirms quantitatively that most of the chemical energy derived from the photons absorbed by PSII does not end up in the hydrogen carrier produced, PQH_2_, but in O_2_.

### 3.8. Photosynthetic Efficiency: Is It Meaningful?

It is tempting to try to quantify the efficiency of photosynthesis, for instance by calculating the ratio of the free-energy increase during glucose production according to Equation (1) to the total photon energy absorbed by the leaf or the total solar energy impinging on the leaf [26,36]. We propose in the following that from biological and chemical-energy standpoints the analysis may be misguided due to incorrect assumptions about the function of photosynthesis, its reactions, and where the photon energy is stored.

From a biological perspective, the primary function of photosynthesis is not energy conversion but synthesis of biomolecules, as the term photosynthesis properly indicates. For instance, trees produce wood not to store chemical energy but to expose leaves in a broad canopy to direct sunlight, keep them out of the reach of large herbivores, etc. In fact, most of the chemical energy produced in oxygenic photosynthesis is stored not in biomass but in O_2_ with its relatively weak double bond [15]. The examples of algal blooms and desert plants show that scarcity of nutrients or water, rather than of photon energy, often limit plant growth, so optimization of photosynthetic efficiency may not be critical to the plant.

Quantitative analyses of the efficiency of photosynthesis mostly focus on glucose production according to Equation (1). They overlook that most of the chemical energy is stored in the co-product O_2_ [3,15] and, therefore, does not benefit the plant directly, and that photosynthesis can occur without glucose production, producing ATP for the organism according to Equation (3), e.g., using cyclic electron transport. This means that the result of the traditional efficiency calculation may not be really meaningful in a bioenergetics context.

### 3.9. Challenging the Paradigm of Electron Transport as Energy Transport

Traditional explanations of photosynthesis, most notably the Z-scheme, as well as of aerobic respiration have emphasized electron transport and equated it with energy flow, implicitly by red-hot electrons shown flowing through a cell [2] or explicitly when “high-energy electrons” [16,18,34,43] are invoked. According to a widely held view exemplified by Goodsell [43], photosynthesis “uses the energy of … photons to create high-energy electrons”, which are “used to power the pumping of hydrogen ions across the membrane” or transferred to NADPH. This interpretation is encapsulated in the widely quoted aphorism: “What drives life is thus a little electric current, kept up by the sunshine” [44]. In the following sections, these concepts are discussed critically, confirming the conclusion that what really drives complex life is chemical energy stored in relatively weak bonds, mostly in O_2_ [3], kept up by the sunshine.

#### 3.9.1. No “High-Energy Electrons” in Photosynthesis

The idea that photons produce high-energy electrons does not stand up to critical analysis. For instance, Figure 1 and chemical intuition shows that the unstable radical P680^+^˙ generated with the help of photon energy is energy-rich, but it is electron-*deficient* compared to P680 and P680*. Therefore, P680^+^˙ does not release a putative high-energy electron; in fact, it is an electron *acceptor*.

The misguided concept of “high-energy electrons” is linked by the erroneous “electron waterfall” [18,34] interpretation of a step in the Z-scheme as a high-energy donor passing an electron to a low-energy acceptor. In fact, the free energy released during a redox reaction represented by a step in the Z-diagram depends on the free energies of *two* donor and *two* acceptor species, as shown in the SI, and it is larger in magnitude the *higher* the energy of the actual acceptor of the electron (Equation (S52)). The absurdity of the traditional “electron waterfall” interpretation becomes apparent when one applies it to the left end of the Z-scheme in Figure 6 and Figure S1, where it leads to the unreasonable proposition that electrons flow spontaneously from H_2_O via the OEC and tyrosine to ground-state chlorophyll and release energy in the process.

To the right of each photosystem, the implications of the conventional Z-scheme are unclear. For instance, it suggests (see Figure S1) spontaneous, energy-releasing electron transfer from Pheo to Q_A_ and then Q_B_. However, Pheo is an electron acceptor, not an electron donor, so the suggested electron transfer does not occur between these species. The EZ-scheme is much superior in providing a self-explanatory diagram of energy absorption, transfer and release, and the associated electron transport in photosynthesis. It shows the relative free energies of all the molecules and ions involved, which reflect not nebulous electron affinities but bond, ionization, and solvation energies—in other words, chemical energy of molecules and ions [32].

Finally, high-energy electrons are supposedly transferred to NADPH; [45,46] this is often invoked to explain the large heat release (comparable with H_2_ on a molar basis) when NADH, a close analogue of NADPH, reacts with O_2_ [3]. However, this misassigns the energy of O_2_ to NADH [3]. Analyzing other reactions of NAD(P)H, we have shown that it is only of moderate energy [3].

#### 3.9.2. Energy Flow Runs Counter to Electron Flow

We had previously pointed out that electron flow runs *opposite* to energy flow in electron-transport chains [3]. Figure 5, as well as a careful analysis of Figure 1, Figure 2, Figure 3 and Figure 4, confirms that while electrons flow from left to right, energy flows from right to left (and up when photons are absorbed). For instance, energy clearly flows from high-energy, unstable P680^+^˙ to the left to split low-energy, stable H_2_O, while electrons flow to the right. In any of the redox pairs without photon energy input in the Z- or EZ-scheme, the reduction, on the right, releases more free energy than the oxidation, to the left, takes up, so energy arguably flows from right to left. For instance, P680^+^˙ transfers energy into Tyr_Z_˙ + H^+^, from right to left.

Figure 1, Figure 2, Figure 3, Figure 4 and Figure 5, in agreement with basic chemical intuition, show that electron-carrying (reduced) species, such as stable P680 and Tyr_Z_, are generally *lower* in energy than their oxidized counterparts, such as the electron-deficient, high-energy, unstable radicals P680^+^˙ and Tyr_Z_˙ + H^+^. Similarly, ionization of Cu^+^ to Cu^2+^ in plastocyanin or of Fe^2+^ to Fe^3+^ in ferredoxin requires energy input but *removes* an electron. Thus, it is the net movement of oxidized, high-energy species, which are *deficient* in electrons, that generally represents energy flow. This confirms that the energy transferred is not carried by the transported electrons.

#### 3.9.3. Hydrogen Transfer, Not Electron Transport, as the Main Function of Photosynthesis

At and above the college level, photosynthesis has traditionally been explained in terms of electron transport [2,9,18,43], but it is not always clarified explicitly why an organism needs electron transport. We argue that the main function of photosynthesis is the (formal) transfer of hydrogen atoms from water to carbon dioxide in order to form carbohydrates (CH_2_O)_n_ and other biomolecules (photosynthesis). As highlighted at the bottom of Figure 4 and Figure 5, in PSII, H atoms are effectively (i.e., formally) transferred from H_2_O into PQH_2_, in PSI from PQH_2_ into NADPH + H^+^, and in the Calvin cycle from 3 (NADPH + H^+^) into the three-carbon sugar glyceraldehyde 3-phosphate, C_3_H_5_O_3_PO_3_H. In many steps along this path, instead of an actual H-atom, H^+^ and e^−^ are transferred separately. Thus, distinct electron transport does occur, but only locally, over short distances within the thylakoid membrane. On a larger scale, H-atoms bound in molecules or ions of various oxidation states are transported in photosynthesis, not electrons as in a wire. Diffusion of plastoquinol, plastocyanin, or ferredoxin is not associated with any definable electron current. In the cell medium, electrical current is usually carried by protons and other ions, not by distinct electrons.

#### 3.9.4. The Bicycle Chain: An Analogy for Electron Transport

Electron flow is often taken as an implicit marker of a series of spontaneous redox processes. It is sometimes said that a reaction, or in the quote above life itself, is driven by electron transport. This is as true as saying that a bicycle is propelled by its chain. Just like the chain is actually driven by the pedaling cyclist, the electrons are driven by the free-energy difference between high-energy reactants and low-energy products, which are often due to differences in bond energies—in short, chemical energy [15,32].

In this author’s view, the transfer of hydrogen to CO_2_ to form biomolecules is the main function of photosynthesis, and electron transfer is only a means to that end. To present electron transport as the functional purpose of photosynthesis is like saying that the purpose of a bicycle ride is to move the chain, rather than to transport the rider.

### 3.10. Synopsis: A Valid Description of Energy and Hydrogen Flow in Photosynthesis

Casting aside historical misconceptions, the flow of energy, electrons, and hydrogen in photosynthesis can be cogently summarized based on the EZ-scheme as shown in Figure 4 and Figure 5: Photon energy excites the chlorophyll dimer P680 in Photosystem II to P680*, whose loosened electron can be taken up by pheophytin, a chlorophyll without Mg. It converts to pheophytin^−^˙ and gives off the LUMO binding energy of the added electron, turning P680* into the high-energy, electron-deficient radical P680^+^˙ (charge separation). Unstable P680^+^˙ quickly returns to the ground state, P680, by ripping an electron off tyrosine_Z_, breaking an O–H bond and generating the unstable, high-energy Tyr_Z_˙ radical and a proton. Tyr_Z_˙ recovers its electron from H_2_O by means of the Mn_4_CaO_5_ water-splitting complex. The energy released by P680^+^˙ returning to ground-state P680 four times is used to split 2 H_2_O and generate O_2_, a high-energy molecule, after four photons have been absorbed. Returning to pheophytin^−^˙, we find that its additional electron is taken up by an even more avid electron acceptor, PSII-bound quinone Q in site A, to form Q_A_^−^˙ and then by quinone Q in site B, which in two such steps with two protons forms stable Q_B_H_2_. Overall in PSII, four H-atoms have been formally transferred from 2 H_2_O to 2 Q_B_H_2_, a reluctant hydrogen donor that cannot generate carbohydrates, (CH_2_O)_n_, from CO_2_, while photon energy has been stored in the weak double bond of O_2_.

For this process to occur again, Q_B_ has to be recovered from Q_B_H_2_. This is achieved with photon energy absorbed by P700 in PSI and transferred to plastoquinol PQH_2_, the free form of Q_B_H_2_, via P700^+^˙, diffusing soluble plastocyanin, cytochrome *b*_6_*f* and its Rieske protein. The electrons taken from Q_B_H_2_ are transported in the opposite direction between these species. Through coupled redox reactions of 2 PQH_2_ and the Rieske protein, protons are pumped through the thylakoid membrane, storing some of the photon energy in a proton gradient that drives ATP synthesis. The electron given off in the formation of P700^+^˙ is taken up by the acceptor A_0_. Since P700* is easier to ionize than P680*, A_0_ does not need to release as much energy as pheophytin when it accepts the electron. After several electron-transport redox steps, which include soluble ferredoxin that can diffuse to ferredoxin–NADP^+^ reductase, this allows for the reduction of NADP^+^ to NADPH + H^+^, a biochemical analogue of H_2_ that gives off hydrogen atoms more easily upon oxidation than does PQH_2_. This allows NADPH + H^+^ to transfer hydrogen to CO_2_ in the Calvin cycle and form carbohydrates, (CH_2_O)_n_, helped along by hydrolysis of several ATP molecules.

Summarizing the essential points even more succinctly: photon absorption in PSII helps generate the high-energy electron acceptor P680^+^˙, which can split water to form high-energy O_2_ while H-atoms are in effect transferred (as protons plus electrons) to form plastoquinol. The latter subsequently performs proton transport for ATP synthesis driven by energy passed down from light-generated, electron-deficient P700^+^˙ generated in PSI. Electrons given off in the formation of P700^+^˙ eventually combine with NADP^+^ and H^+^ to form NADPH, which in turn transfers H atoms to CO_2_ to form carbohydrates in the Calvin cycle.

## 4. Conclusions

In this paper, it has been highlighted that oxygenic photosynthesis in plants does not produce high-energy organic molecules or high-energy electrons, but has two other functions: (i) making hydrogen from H_2_O available for bonding to CO_2_ and thus synthesis of carbohydrates and other biomolecules; (ii) converting solar energy into chemical energy stored in ATP. Step (i) requires production of the unavoidable high-energy “waste product” O_2_. The photon energy absorbed by Photosystem II is essentially used up to produce O_2_. Step (ii) includes “cyclic electron transport”, which is known to not involve carbohydrate but only chemical-energy production. The new analysis explains convincingly why plants need two photosystems: (i) PSII to extract hydrogen from H_2_O, generating not only the low-energy hydrogen carrier PQH_2_ but also the energetically expensive product O_2_; this step provides little energy directly to the plant; (ii) PSI to produce stored chemical energy and eventually ATP as well as a hydrogen carrier of moderate free energy, NADPH. Fully consistent with our analysis, anoxygenic photosynthesis, e.g., transferring hydrogen from H_2_S or H_2_ to CO_2_ without O_2_ production, requires only one photosystem. Important aspects of oxygenic photosynthesis, e.g., charge separation by generation of Pheo^−^˙ and high-energy P680^+^˙, which had previously been described only in words, have been represented in an energy diagram for the first time. Others, such as the major energy boost from 680* to P680^+^˙ provided by the “electron affinity” (LUMO binding energy) of Pheo, have been completely missing from textbooks. That the crucial difference in the ionization energies of P680* and P700* results in P700^+^˙ having too little energy to split water and generate O_2_ has also not been much emphasized. Unlike the traditional Z-scheme, which encodes energy differences on a shifted scale and in volts, the comprehensive EZ-scheme of photosynthesis introduced here shows free-energy differences explicitly, in kJ/mol. This is made possible by the use of individual free energies in this work, while conventional electrochemical thermodynamics, due to its arbitrary choice of the zero point of energy, can only predict *relative* redox energy *differences*. Our analysis has pointed out that the Z-scheme as traditionally presented shows O_2_ incorrectly as a low-energy species, does not explain why electrons move from H_2_O into tyrosine and chlorophyll, misses important high-energy species, and does not include the Kok and Calvin cycles. All these shortcomings have been addressed in the self-explanatory EZ-scheme, which also documents the counterflow of electrons and energy in photosynthesis and thus disproves the notion of high-energy electrons transporting energy.

## Supplementary Materials

The following are available online at https://www.mdpi.com/article/10.3390/life11111191/s1, Figure S1: Z-scheme of photosynthesis; list of redox species in photosynthesis; shortcomings of the Z-scheme; Figure S2: overview of heat transfer; entropy change in an endergonic reaction and in photon emission & absorption; electron acceptors of high energy; Figure S3: redox energy levels; Figure S4: hydrogen energies; free energies and redox potentials; Figure S5: voltages in redox reactions and galvanic cells; Figure S6: series of galvanic cells mimicking the photosynthetic electron transport chain; Figure S7: Z-scheme with pure energy transfer; Figure S8: EZ-scheme with pure energy transfer; Figure S9: cyclic electron transport; energetics of CO_2_; Figure S10: comprehensive EZ-scheme; Figures S11 & S12: alternative EZ-schemes.

## Abbreviations

A_0_	An acceptor near P700 in PSI
A_0_^−^˙	Acceptor A_0_ after it has taken up an electron; a radical anion
A_1_	Phylloquinone, also known as phytomenadione, a fat-soluble naphthoquinone derivative in PSI
[CH_2_O]	Generic carbohydrate
Cyt*f*	Cytochrome *b_6_f*, a dimeric enzyme in the thylakoid membrane in a chloroplast
Fd	Ferredoxin, a soluble iron–sulfur protein on the stroma side of the thylakoid membrane, containing a [Fe_2_S_2_] cluster
Fe^2+^ in [Fe_2_S_2_] (Rieske)	Reduced form [Fe^2+^Fe^3+^S_2_] of the [Fe_2_S_2_] or 2Fe-2S cluster in a Rieske iron–sulfur protein
[Fe_4_S_4_]	Iron–sulfur clusters, also denoted as 4Fe-4S, in PSI
FNR	Ferredoxin–NADP^+^ reductase (or ferredoxin:NADP^+^ oxidoreductase), an enzyme catalyzing the reduction (“hydrogenation”) of NADP^+^ coupled with the oxidation of reduced ferredoxin
NADP^+^	Nicotinamide adenine dinucleotide phosphate
NADPH	The reduced form of NADP^+^; NADPH + H^+^ is a slightly lower-energy biochemical analogue of H_2_ [3]
P680	PSII primary electron donor, a pigment (special chlorophyll dimer) with an absorption maximum near a wavelength of 680 nm
P680*	The electronically excited state of P680 after photon absorption
P680^+^˙	P680* after loss of an electron; a radical cation; the oxidized counterpart of both P680 and P680*
P700	PSI primary electron donor, the reaction-center chlorophyll-*a* dimer, with an absorption maximum near 700 nm
P700*	The electronically excited state of P700 after photon absorption
P700^+^˙	P700* after loss of an electron; a radical cation; the oxidized counterpart of both P700 and P700*
PC	Plastocyanin, a soluble protein with a redox-active copper ion, on the lumen side of the thylakoid membrane
Pheo	Pheophytin (chlorophyll without the Mg^2+^ ion) near P680
Pheo^−^˙	Pheophytin that has taken up an electron; a radical anion
PQ	Free plastoquinone, a benzoquinone derivative similar to ubiquinone (coenzyme Q)
PQH_2_	Plastoquinol, the hydrogenated (fully reduced) form of PQ
PSI	Photosystem I, a protein complex in the thylakoid membrane
PSII	Photosystem II, a protein complex in the thylakoid membrane
Q_A_ or PQ-A	Protein-bound plastoquinone near pheophytin in PSII
Q_A_^−^˙	Q_A_ that has taken up an electron; a radical anion
Q_B_ or PQ-B	Loosely bound plastoquinone in PSII
Q_B_H_2_	The hydrogenated (fully reduced) form of Q_B_
Rieske Fe^3+^ in [Fe_2_S_2_]	Oxidized form [Fe^3+^_2_S_2_] of the [Fe_2_S_2_] cluster in a Rieske protein
S_0_–S_4_	States of the Mn_4_CaO_5_ water-splitting complex (oxygen-evolving complex, OEC) and associated H_2_O molecules, of increasing oxidation number and energy, in the S-cycle or Kok cycle
Tyr_Z_	A tyrosine residue near P680 in PSII
Tyr_Z_˙	Tyr_Z_ after removal of the hydrogen (H^+^ + e^−^) from the OH group; a radical
