# Supplementary material for: O2 and Other High-Energy Molecules in Photosynthesis: Why Plants Need Two Photosystems
Source: Life (Basel). 2021 Nov 5;11(11):1191. doi: 10.3390/life11111191 (PMC8621363; doi:10.3390/life11111191)
Supplement: Supplementary file 1 [file life-11-01191-s001.zip › life-1345305-supplementary.pdf]

Supporting Information

for

O_2 and Other High-Energy Molecules in Photosynthesis: Why Plants Need Two Photosystems

Klaus Schmidt-Rohr*

Department of Chemistry, Brandeis University, Waltham MA 02465, USA

Life, 2021

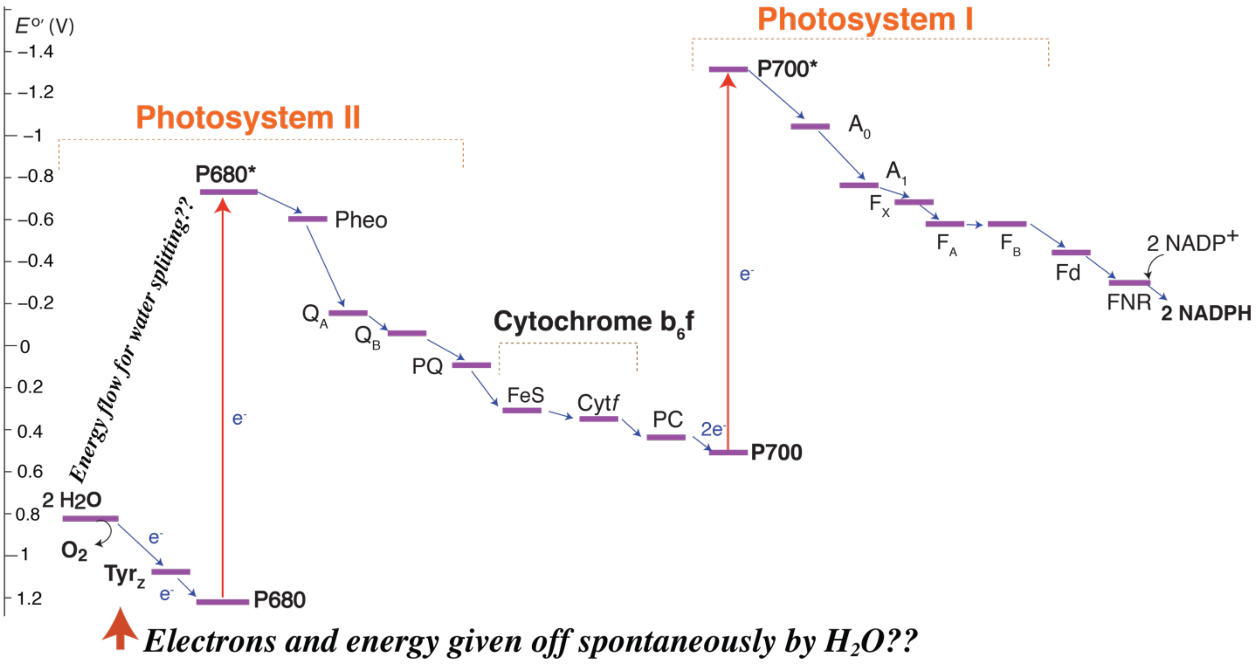

Figure S1. Z-scheme of photosynthesis in plants as shown in many textbooks [1-5] and similar as in the specialized literature [6]. The vertical axis shows (negative) standard reduction potentials. The energy flow from P680* to H_2O needed to achieve water splitting is not apparent. The spontaneous flow of electrons and energy out of H_2O counterfactually suggested by the scheme interpreted in terms of the electron-waterfall concept [3, 7] is highlighted in the lower left corner.

List of redox species in the Z-scheme of photosynthesis. The following is a list and brief description of species undergoing redox reactions in photosynthesis in plants, in the order in which they appear in the Z-scheme, see Figure S1. The corresponding alphabetical listing can be found at the end of the main text. For the chemical structures of these molecules, which are secondary to our energetic analysis, the reader should consult the specialized literature.

PSII: Photosystem II, a protein complex in the thylakoid membrane in a chloroplast

$O_2 + 4H^+$: Molecular oxygen plus four hydrated protons

$S_0 – S_4$: States of the Mn_4CaO_5 water-splitting complex (oxygen-evolving complex, OEC) and associated H_2O molecules, of increasing oxidation number and energy, in the S-cycle or Kok cycle

Tyr_Z: A tyrosine residue near P680 in PSII

$Tyr_Z^{\cdot}$: Tyr_Z after removal of the hydrogen ($H^+ + e^-$) from the OH group; a radical

P680: PSII primary electron donor, a pigment (special chlorophyll dimer) with an absorption maximum near a wavelength of 680 nm

P680*: The electronically excited state of P680 after photon absorption

$P680^{+\cdot}$: P680* after loss of an electron; a radical cation; the oxidized counterpart of both P680 and P680*

Pheo: Pheophytin (chlorophyll without the Mg^{2+} ion) near P680

$Pheo^{-\cdot}$: Pheophytin that has taken up an electron; a radical anion

Q_A or PQ-A: Protein-bound plastoquinone near pheophytin in PSII

$Q_A^{-\cdot}$: Q_A that has taken up an electron; a radical anion

Q_B or PQ-B: Loosely bound plastoquinone in PSII

Q_BH_2: The hydrogenated (fully reduced) form of Q_B

PQ: Free plastoquinone, a benzoquinone derivative similar to ubiquinone (coenzyme Q)

PQH_2: Plastoquinol, the hydrogenated (fully reduced) form of PQ

Cyt*f*: Cytochrome b_6f, a dimeric enzyme in the thylakoid membrane

FeS, or Fe^{2+} in $[Fe_2S_2]$ (Rieske): Reduced form $[Fe^{2+}Fe^{3+}S_2]$ of the $[Fe_2S_2]$ or 2Fe-2S cluster in a Rieske iron–sulfur protein

Rieske Fe^{3+} in $[Fe_2S_2]$: Oxidized form $[Fe^{3+}{}_2S_2]$ of the $[Fe_2S_2]$ cluster in a Rieske protein

PC: Plastocyanin, a soluble protein with a redox-active copper ion, on the lumen side of the thylakoid membrane

PSI: Photosystem I, a protein complex in the thylakoid membrane

P700: PSI primary electron donor, the reaction-center chlorophyll-*a* dimer, with an absorption maximum near 700 nm

P700*: The electronically excited state of P700 after photon absorption

$P700^{+\cdot}$: P700* after loss of an electron; a radical cation; the oxidized counterpart of both P700 and P700*

A_0: An acceptor near P700 in PSI

$A_0^{-\cdot}$: Acceptor A_0 after it has taken up an electron; a radical anion

A_1: Phylloquinone, also known as phytomenadione, a fat-soluble naphthoquinone derivative in PSI

F_X, F_A, F_B: [Fe_4S_4] iron–sulfur clusters, also denoted as 4Fe-4S, in PSI

Fd: Ferredoxin, a soluble iron–sulfur protein on the stroma side of the thylakoid membrane, containing a [Fe_2S_2] cluster

FNR: Ferredoxin–$NADP^+$ reductase (or ferredoxin:$NADP^+$ oxidoreductase), an enzyme catalyzing the reduction ("hydrogenation") of $NADP^+$ coupled with the oxidation of reduced ferredoxin

$NADP^+$: Nicotinamide adenine dinucleotide phosphate

NADPH: The reduced form of $NADP^+$; NADPH + H^+ is a slightly lower-energy biochemical analogue of H_2 [8]

[CH_2O]: Generic carbohydrate

Shortcomings of the traditional Z-scheme

A representation of the energetics of photosynthesis in terms of the Z-scheme of standard reduction potentials associated with Photosystems I and II can be found in most biochemistry textbooks [1-6], specialized literature [9-11] and Figure S1. The various redox-active species involved are listed and briefly described further below. The traditional Z-scheme summarizes many useful energetic data of redox reactions in photosynthesis but it also has several shortcomings. It

- shows O_2 at low, rather than correctly at high energy;
- misses other important high-energy species such as $P680^{+\cdot}$, "the strongest biological oxidizing agent known" (Wikipedia);
- does not show where the energy for water splitting comes from, since no energy flow from excited P680 to H_2O is apparent;
- does not show charge separation;
- is inconsistent, switching from showing reduced species (H_2O, Tyr_Z, P680) to showing oxidized species (Pheo, Q_A);
- does not explain why PSI has insufficient energy to split water;
- does not explain why P700 is shifted up relative to P680;
- does not explain why plants need two photosystems;
- suggests that electrons spontaneously move from H_2O to Tyr and then ground-state chlorophyll, surprisingly releasing energy, all before photon absorption;
- does not show that 2×4 photons are needed per O_2 produced;
- makes pure energy transfer (e.g. from P680* to a neighboring chlorophyll) [12] look disjointed (see Figure S7);

- shows energies in volts, rather than kJ/mol;
- is easily misinterpreted as a diagram of the energies of chemical species[9, 11] rather than *differences* between the energies of *two* or more species in a half reaction (see below);[8]
- does not even show meaningful energy differences (see below);
- commonly shows $NADP^+$ and NADPH at different standard reduction potentials.[1, 3, 4]

There is no evidence of an underlying higher-level description, e.g., by the researchers who developed the Z-scheme, of the role of $P680^{+\cdot}$ or the high energy of O_2, see the SI. Neither is mentioned as a high-energy species in a recent review of the development of the Z-scheme.[6] This is also indicated when the developers of the Z-scheme write that "P680* recovers its electron" while the electron is really recovered by $P680^{+\cdot}$, which is the form of P680 that is an electron acceptor, or maybe by P680, which is recovered when the electron has been recovered, but not by P680*, which must be bypassed by any recovery after it has lost its electron.

Some of the shortcomings in the traditional Z-scheme can be directly corrected, as shown below, but others cannot be fully rectified in the framework of standard reduction potentials. All the listed shortcomings are eliminated in an expanded Z-scheme based on redox energy transfer and release diagrams [8], as explained in the main text.

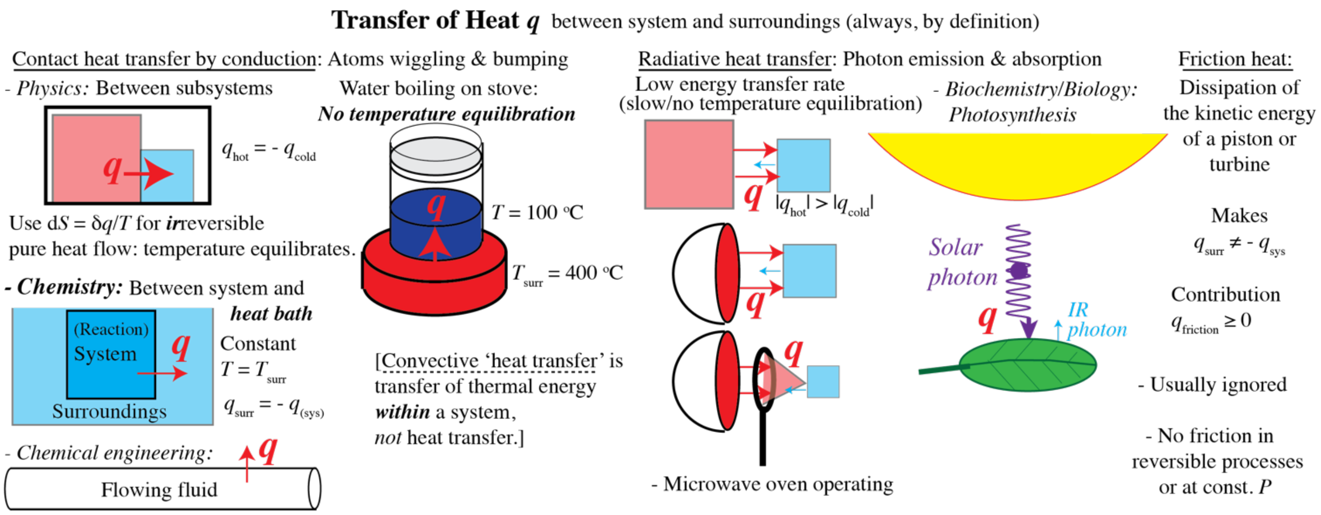

Figure S2. Overview of heat transfer in physics, chemistry, chemical engineering, and biology. Radiative heat transfer by solar photons shown prominently right of center is the starting point of photosynthesis.

Solar energy is heat. According to the first law of thermodynamics, the change in the internal energy U of a closed system, which is the sum of the kinetic and potential energies of the system's molecules, is due to work w and heat q:

$$\Delta U = w + q \qquad \text{(S1)}$$

with the sign convention universally used in chemistry. Atoms cannot enter or leave a closed system as defined in chemistry, but energy exchange is possible. Work is generated by a force acting through a macroscopic distance. Any other form of energy transferred between a closed system and its surroundings is heat. While heat transferred through contact between bodies of different temperature is most commonly discussed in basic thermodynamics, radiative heat transfer or friction heating (see Figure S2) can also be important. Light energy from the Sun, well approximated as a blackbody is clearly in the category of radiative heat,[13] and it is treated as such in the thermodynamic analysis of photosynthesis outlined in the following. Thus, the solar photons driving photosynthesis are heat, which disproves a textbook statement that "Heat flow is not a source of energy for cells".[1] Any treatment[14] that refers to work performed during light absorption in photosynthesis needs to be reconsidered in light of the fundamental eq.(S1).

Since the change in system enthalpy at constant pressure equals heat, the enthalpy of the reaction associated with the light absorption can be equal to the photon energy if dissipative heat loss $q_{\mathrm{diss}} < 0$ is negligible:

$$\Delta_{\mathrm{r}}H = q_{\mathrm{P}} = E_{\mathrm{photon}} + q_{\mathrm{diss}} \leq E_{\mathrm{photon}}. \qquad \text{(S2)}$$

In the following long analysis, an analogous upper limit for the free energy $\Delta_{\mathrm{r}}G$ of the light-absorbing reaction is derived from first principles, including the entropy change in photon absorption. In this process, infinitesimally small thermodynamic quantities need to be analyzed. As an example, the first law would be written as

$$\mathrm{d}U = \delta w + \delta q \qquad \text{(S3)}$$

with dU as the infinitesimal change in internal energy U, as state function; infinitesimal work δw (not a change of any state function); and infinitesimal heat δq.

How a greatly endergonic reaction can occur. The second law of thermodynamics in chemistry states categorically that an endergonic reaction (i.e., with $\Delta_{\mathrm{r}}G^{\mathrm{o}} > 0$) will not occur in a closed system at constant T and P, unless driven by non-mechanical work. However, as pointed out above, the photon energy transferred in photosynthesis is not work but radiant heat. The

initial endergonic reaction from P680 to P680* can occur because the transfer of heat from the Sun's surface at 5800 K to the leaf at 300 K is not a constant-temperature process.

Entropy change in a quasistatic reaction. Since photosynthesis involves heat exchange between a system (the leaf) and its surroundings (the Sun) of vastly different temperatures by photon emission and absorption, simple thermodynamics at constant T with heat transfer via direct contact does not necessarily apply. Therefore, we build the analysis up from first principles.

Using the basic equality of Clausius (part of the second law of thermodynamics),

$$\mathrm{d}S = \delta q/T \tag{S4}$$

for a reversible process involving a closed system and the well-known infinitesimal reversible expansion work $\delta w = -P\,\mathrm{d}V$, we can eliminate δq from the first law:

$$\mathrm{d}U = \delta w + \delta q = -P\,\mathrm{d}V + T\,\mathrm{d}S \tag{S5}$$

for a reversible process involving a closed system.

Comparison with the mathematical expression of the exact or complete differential

$$\mathrm{d}U = (\partial U/\partial V)_{S,n}\,\mathrm{d}V + (\partial U/\partial S)_{V,n}\,\mathrm{d}S \tag{S6}$$

gives us partial derivatives:

$$(\partial U/\partial V)_{S,n} = -P\text{: At constant } S \text{ and } n,\ \delta q = 0 \text{ and } \mathrm{d}U = \delta w = -P\,\mathrm{d}V \tag{S7a}$$

$$(\partial U/\partial S)_{V,n} = T\text{: At constant } V,\ \delta w = 0 \text{ and } \mathrm{d}U = \delta q = T\,\mathrm{d}S \text{ (at constant } n) \tag{S7b}$$

We derive the fundamental relation from the fact that U is a state function and therefore according to multivariable calculus the exact (or complete, or total) differential dU can be written as

$$\mathrm{d}U = (\partial U/\partial V)_{S,n}\,\mathrm{d}V + (\partial U/\partial S)_{V,n}\,\mathrm{d}S + \Sigma_i\,(\partial U/\partial n_i)_{S,V,n'}\,\mathrm{d}n_i. \tag{S8}$$

Using the partial derivatives from eq.(S7) and defining the chemical potential of substance i

$$(\partial U/\partial n_i)_{S,V,n'} = \mu_i \tag{S9}$$

we obtain the fundamental relation of chemical thermodynamics

$$dU = - P\, dV + T\, dS + \Sigma_i\, \mu_i\, dn_i. \qquad \text{(S10)}$$

Note that this applies (only) for processes with well-defined P and T: uniform P & T, "uPT" processes (see J. Chem. Educ. 91, 402, 2014). One can show that the chemical potentials μ_i are the partial molar Gibbs free energies, i.e., that $G = \Sigma_i\, \mu_i\, n_i$.

We use $\delta w = - P\, dV$ for a quasistatic process in a closed system (see J. Chem. Educ. 91, 402, 2014) to get

$$dU = - P\, dV + \boldsymbol{\delta q}. \qquad \text{(S11)}$$

Comparison with the fundamental relation $dU = - P\, dV + \boldsymbol{T\, dS + \Sigma_i\, \mu_i\, dn_i}$
shows that

$$\boldsymbol{\delta q = T\, dS + \Sigma_i\, \mu_i\, dn_i} \qquad \text{(S12)}$$

for a quasistatic process without electrical work in a closed system.

This gives us the Clausius-De Donder equality[15]

$$\boldsymbol{dS = \delta q/T - \Sigma_i\, \mu_i\, dn_i/T} \qquad \text{(S13)}$$

for the entropy change due to a chemical reaction without fast mechanical processes (e.g., at const. P or V) and without electrical work at a system temperature T (which was not assumed to be constant).

For pure heat flow, which excludes composition changes, i.e., with $dn_i = 0$, the equation simplifies to

$$\boldsymbol{dS = \delta q/T} \quad \text{(pure heat flow)} \qquad \text{(S14)}$$

even if the heat flow is irreversible, see also J. Chem. Educ. 91, 402, 2014.

Since

$$dG = \Sigma_i\, \mu_i\, dn_i \text{ at constant } T \text{ and } P, \qquad \text{(S15)}$$

under these conditions we can integrate eq.(S13) to obtain a related but simpler-looking expression,

$$\Delta S = q/T - \Delta_r G/T. \qquad \text{(S16)}$$

This relation can also be obtained from

$$\Delta G = \Delta H - T\,\Delta S \text{ at constant } T \text{ and } P, \qquad \text{(S17)}$$

with $\Delta H = q_P$, solved for

$$\Delta S = \Delta H/T - \Delta G = q/T - \Delta G/T. \qquad \text{(S18)}$$

However, eq.(S16/18) is mostly a conceptual and notational simplification under restrictive conditions. The more complicated eq.(S13) applies more generally, specifically without requiring constant T.

Entropy changes in photon emission and absorption. The radiative heat transfer of interest in photosynthesis is a two-step process of solar photon emission and absorption, which makes it more complicated than the direct heat transfer between subsystems in contact as usually analyzed in introductory thermodynamics. When heat is transferred between a hot and a cold body in contact, the entropy of the hot body can *de*crease spontaneously because this reduction is more than compensated by the simultaneous entropy increase in the cold body. In radiative heat transfer, this is not the case. For instance, the entropy decrease of the un due to emission of a photon occurs 8 minutes before the entropy increase in the leaf due to absorption of the solar photon. In the following, we review how an increase in total entropy occurs in both steps of radiative heat transfer, photon emission and absorption.

Entropy change due to solar-photon emission. First, we consider emission of solar photons of energy E_{photons} (heat given off by the sun, apart from the sign). The entropy of the sun decreases according to

$$\Delta S_{\text{sys}} = \Delta S_{\text{sun}} = q_{\text{solar}}/T_{\text{sun}} = - E_{\text{photons}}/T_{\text{sun}} < 0. \qquad \text{(S19)}$$

Here we used that the equality of Clausius, $dS = \delta q/T$, applies not only to reversible processes but also to irreversible pure heat transfer, see eq.(S14). The entropy reduction in eq.(S19) is overcompensated by an entropy increase in space[13, 14, 16, 17] due to the added photons that amounts to

$$\Delta S_{\text{surr}} = \Delta S_{\text{space}} = 4/3\ E_{\text{photons}}/T_{\text{sun}}, \qquad \text{(S20)}$$

so the total entropy increase is positive:

$$\Delta S_{\text{tot}} = \Delta S_{\text{sys}} + \Delta S_{\text{surr}} = 1/3\ E_{\text{photons}}/T_{\text{sun}} > 0. \qquad \text{(S21)}$$

The crucial factor 4/3 in eq.(S20) comes from the theory of thermal photon emission,[13, 16, 18, 19] commonly referred to as blackbody radiation. According to the Stefan-Boltzmann law, the total photon energy given off by the thermal emitter scales with fourth power of temperature,

$$E_{\text{photons}} = \sigma\, T^4 \qquad \text{(S22)}$$

while the total entropy of the emitted photons is [13, 16, 19]

$$S_{\text{photons}} = 4/3\ \sigma\, T^3 = 4/3\ E_{\text{photons}}/T. \qquad \text{(S23)}$$

The proportionality constant σ is similar as given in the literature [19] but its value is not needed here. Equation (S23) is consistent with the typical photon energy [19]

$$E_{\text{photon}} = \langle E_{\text{photons}}\rangle/\langle N_{\text{photons}}\rangle = 2.7\ kT \qquad \text{(S24)}$$

and the typical photon entropy

$$S_{\text{photon}} = \langle S_{\text{photons}}\rangle/\langle N_{\text{photons}}\rangle = 3.6\ k = 4/3\ E_{\text{photon}}/T. \qquad \text{(S25)}$$

Entropy change during solar-photon absorption. In a proper analysis of absorption by the leaf, it must be taken into account that the loss of the solar photon heat energy and entropy from space, - 4/3 $E_{\text{solar}}/T_{\text{sun}}$, is partly offset by thermal radiation (IR photons) emitted by the solar-photon absorber (the leaf). This ensures an increase in total entropy even when the emitter and absorber temperatures are similar. Specifically, also considering the emission by the colder body prevents the theory from predicting spurious one-way heat transfer between two bodies of equal temperature.

Solar photons of energy $E_{\text{solar}} = \sigma\, T_{\text{sun}}^4$ and entropy $S_{\text{solar}} = 4/3\ \sigma\, T_{\text{sun}}^3$ are absorbed at T_{leaf}, while IR photons of energy $E_{\text{leaf-IR}} = \xi\, \sigma\, T_{\text{leaf}}^4$ and entropy $S_{\text{leaf-IR}} = 4/3\ \xi\, \sigma\, T_{\text{leaf}}^3$ are emitted. The unitless factor $\xi \geq 1$ can account for the larger solid angle of the outgoing IR relative to the incoming solar radiation. An analysis based on exergy [16] corresponds to setting $\xi = 1$. The net heat transfer affecting the leaf is

$$q = E_{\text{solar}} - E_{\text{leaf-IR}} = \sigma\, T_{\text{sun}}^4 - \xi\, \sigma\, T_{\text{leaf}}^4 = E_{\text{solar}}\, [1 - \xi\, (T_{\text{leaf}}/T_{\text{sun}})^4]. \qquad \text{(S26)}$$

The entropy change in the system (the leaf) is

$$\Delta S_{\text{leaf}} = q/T_{\text{leaf}} - \Delta_{\text{r}}G/T_{\text{leaf}} \qquad \text{(S27)}$$

according to the Clausius-De Donder equation (S13) for an entropy change in a chemical reaction (e.g., converting P680 to the higher-energy P680* in Photosystem II) derived above, in its simplified form of eq.(S16), at a system temperature T_{leaf} and pressure P. The term q/T_{leaf} in eq.(S27) with $q > 0$ and small T_{leaf} in the denominator represents a big entropy increase. The entropy change in the surrounding space due to IR-photon emission and solar-photon absorption is

$$\Delta S_{space} = S_{leaf\text{-}IR} - S_{solar} = 4/3\ \sigma\ (\xi\ T_{leaf}^3 - T_{sun}^3) = -\ 4/3\ \sigma\ T_{sun}^3\ (1 - \xi\ (T_{leaf}/T_{sun})^3)$$
$$= -\ 4/3\ E_{solar}/T_{sun}\ (1 - \xi\ (T_{leaf}/T_{sun})^3). \qquad \text{(S28)}$$

where $E_{solar} = \sigma\ T_{sun}^4$ solved for $\sigma = E_{solar}/T_{sun}^4$ was used. Combining the entropy changes in the system and its surroundings (i.e., the leaf and space around it, respectively), we obtain

$$\Delta S_{tot} = \Delta S_{leaf} + \Delta S_{space} > 0 \quad \text{or} \qquad \text{(S29a)}$$
$$q/T_{leaf} - \Delta_r G/T_{leaf} = \Delta S_{leaf} > -\ \Delta S_{space} \quad \text{or} \qquad \text{(S29b)}$$
$$\Delta_r G < q + T_{leaf}\ \Delta S_{space}. \qquad \text{(S29c)}$$

At constant $T = T_{leaf} = T_{sun}$ and if space was a solid body providing contact heat, $T_{leaf}\ \Delta S_{space} = T\ (-q/T) = -q$ would apply and simplify eq.(S29c) to the familiar $\Delta_r G < 0$, but conditions are different here. With q from eq.(S26) and ΔS_{space} from eq.(S28), eq.(S29c) gives an upper limit for the free-energy change in the photochemical reaction:

$$\Delta_r G < E_{solar}\ [1 - \xi\ (T_{leaf}/T_{sun})^4] - 4/3\ E_{solar}\ T_{leaf}/T_{sun}\ (1 - \xi\ (T_{leaf}/T_{sun})^3). \qquad \text{(S30)}$$

The terms on the right-hand side, which go to zero for $T_{leaf} = T_{sun}$ at $\xi = 1$ as required, can be rearranged:

$$\Delta_r G < E_{solar}\ (1 - 4/3\ T_{leaf}/T_{sun} + 1/3\ \xi\ (T_{leaf}/T_{sun})^4) \leq E_{solar}\ (1 - T_{leaf}/T_{sun}) \qquad \text{(S31)}$$

The very last inequality is a comparison with the Carnot efficiency limit for heat engines[19], which applies rigorously for $\xi = 1$.

With $T_{sun} = 5800$ K, $T_{leaf} = 300$ K, and ξ between 1 and 1000, we find

$$\Delta_r G < E_{solar}\ 0.93. \qquad \text{(S32)}$$

This shows that up to 93% of the photon energy can be converted to chemical energy. The maximum in the black-body frequency distribution is at 2.8 kT_{sun} = 1.44 eV, corresponding to a

frequency of 340 THz and a wavelength of 880 nm. Due to nonlinear $\lambda = c/\nu$, it is different (see under Wien's displacement law on Wikipedia) for the wavelength distribution, at 4.9 kT_{sun} = 2.53 eV, corresponding to 500 nm in wavelength. Thus, with a typical solar photon energy of ~2 eV or 200 kJ/mol, eq.(S32) gives

$$\Delta_r G < 200 \text{ kJ/mol} \times 0.93 \qquad \text{(S33a)}$$

$$\Delta_r G < +186 \text{ kJ/mol} \qquad \text{(S33b)}$$

This tells us that $\Delta_r G$ can be positive (up to +185 kJ/mol) in photosynthesis.

Instead of the solar surface temperature T_{sun}, the older literature (e.g. refs.[14, 20] as well as Duysens, Plant Physiol. 34, 210, 1959, and Ross, J. Chem. Phys. 45, 1, 1966) often uses an effective radiation temperature of 1000 K to 1350 K (or even an inadvertently negative "absorber temperature" T_c)[20], chosen to account for scattering and diffuse-light absorption. This results in a lower efficiency factor of ~0.7 between E_{solar} and $\Delta_r G$. However, these considerations are not reflected in more recent publications[13, 16, 19, 21] and in the well-tested Shockley-Queisser (J. Appl. Phys. 32, 510, 1961) theory of solar-cell efficiency, which refer only to the solar surface temperature of >5000 K.

Selective solar-photon absorption. The analysis so far has neglected that absorption of photosynthetically active radiation is limited to wavelengths near 700 nm, which corresponds to $E_{solar} = h\,\nu = h\,c/\lambda$ near 170 kJ/mol. The following calculation includes selective absorption, showing that still most of the solar photon energy can become available as chemical energy. Lower "second law photosynthetically active efficiencies" of 0.338 to 0.368 in the literature [16] are defined differently, in terms of the absorbed to the total integrated solar intensity, and not directly relevant, though the underlying theory and analysis by Delgado-Bonal [16] will be very useful in the following.

We consider that only the visible-light fraction ζ of the solar energy is absorbed by the photosynthetic process and the rest is reflected by the leaf. Integration of the Planck distribution from 400 to 700 nm in wavelength has shown that $\zeta = \eta_{PAR} = 0.37$.[16] The corresponding, similar fraction of entropy is $\zeta_S = r\,\zeta$, with $r = 1.18 \approx 1$ since the spectral distributions of emitted energy and entropy are similar in shape.[16] Solar photons of energy $E_{solar} = \zeta\,\sigma\,T_{sun}^4$ and entropy

$$\boldsymbol{S_{solar} = \zeta_S\, 4/3\, \sigma\, T_{sun}^3 = 4/3\, r\, \zeta\, \sigma\, T_{sun}^{\;3} = 4/3\, r\, E_{solar}/T_{sun}} \qquad \text{(S34)}$$

are absorbed at T_{leaf}. These are the dominant contributions to heat and entropy, highlighted in bold in this section. The IR photons of energy $E_{\text{leaf-IR}} = \sigma\, T_{\text{leaf}}{}^4$ and entropy $S_{\text{leaf-IR}} = 4/3\ \sigma\, T_{\text{leaf}}{}^3$ emitted by the leaf could in principle be neglected since $T_{\text{leaf}} << T_{\text{sun}}$. The net heat transfer is

$$\boldsymbol{q = E_{\text{photon}}} - E_{\text{leaf-IR}} = \boldsymbol{\zeta\, \sigma\, T_{\text{sun}}{}^4} - \xi\, \sigma\, T_{\text{leaf}}{}^4 = E_{\text{photon}}\, (1 - \xi\, T_{\text{leaf}}{}^4/(\zeta\, T_{\text{sun}}{}^4)) \qquad \text{(S35)}$$

where E_{photon} replaces E_{solar}, in order to achieve clarity in the final result, where the absorbed photon energy needs to be distinguished from the total incident solar energy.
The entropy change due to IR-photon emission and solar-photon absorption is

$$\boldsymbol{\Delta S_{\text{space}}} = 4/3\ \sigma\, (\xi\, T_{\text{leaf}}{}^3 - \zeta_S\, T_{\text{sun}}{}^3) = \boldsymbol{-\ 4/3\ r\, E_{\text{photon}}/T_{\text{sun}}}\, (1 - \xi\, T_{\text{leaf}}{}^3/(\zeta\, T_{\text{sun}}{}^3)). \qquad \text{(S36)}$$

Combining the entropy changes in the system and its surroundings, we obtain

$$q/T_{\text{leaf}} - \Delta_r G/T_{\text{leaf}} = \Delta S_{\text{leaf}} > -\ \Delta S_{\text{space}} = 4/3\ r\, E_{\text{solar}}/T_{\text{sun}}\, (1 - \xi\, T_{\text{leaf}}{}^3/(\zeta\, T_{\text{sun}}{}^3)) \qquad \text{(S37a)}$$

$$\boldsymbol{\Delta_r G < E_{\text{photon}}\, [1 - 4/3\ r\, T_{\text{leaf}}/T_{\text{sun}}} + 1/3\ \xi\, T_{\text{leaf}}{}^4/(\zeta\, T_{\text{sun}}{}^4)] = E_{\text{solar}}\ \eta. \qquad \text{(S37b)}$$

Since the spectral distributions of emitted energy and entropy look similar[16] we first neglect their slight difference and set $r = 1$. This gives $\Delta_r G < E_{\text{photon}}\ 0.93$, essentially unchanged from the result without selective absorption, eq.(S31), except that the factor E_{photon} is now the energy of photons of visible light. (Note that this is much higher than the photosynthetic efficiency $\eta_{PAR}{}^{ex} = 0.338$ calculated in the literature,[16] which references $\Delta_r G$ to the total, rather than the absorbed, solar energy input.)

In eq.(S37b), the effect of $r = \zeta_S/\zeta$ in the second term, though small, is actually larger than that of ζ in the third. The difficult integrations of wavelength-dependent S and E from 400 to 700 nm needed to determine r have been performed in the literature.[16] Recognizing that exergy Ex as defined in ref.[16] can be written as

$$Ex = (E_{\text{photon}} - E_{\text{leaf-IR}}) - T_{\text{leaf}}\ \Delta S_{\text{space}} = q - T_{\text{leaf}}\ \Delta S_{\text{space}} \qquad \text{(S38)}$$

in our notation, the first, simple half of our eq.(S37a) becomes

$$\Delta_r G < Ex. \qquad \text{(S39)}$$

Defining the maximum efficiency of conversion of absorbed solar photon energy into chemical energy as η, we can write

$$Ex = \eta\, E_{\text{photon}}. \qquad \text{(S40a)}$$

Comparing the inequality resulting from eqs.(S39) and (S40a),

$$\Delta_r G < E_{\text{photon}}\ \eta \qquad \text{(S40b)}$$

with eq.(S37b) for ξ = 1, we see that the unitless efficiency factor in square brackets in eq.(S37b) is

$$\eta = Ex/E_{\text{photon}}, \qquad \text{(S40c)}$$

to be evaluated over the absorption wavelength range of 400 to 700 nm. It is equal to the ratio of efficiencies $\eta_{PAR}{}^{ex}$ and η_{PAR} given in ref.[16]:

$$\eta = \frac{\int_{400nm}^{700nm} Ex(\lambda)\,d\lambda}{\int_{400nm}^{700nm} E_{solar}(\lambda)\,d\lambda} = \frac{\int_{400nm}^{700nm} Ex(\lambda)\,d\lambda \,/ \int_{0}^{\infty} E_{solar}(\lambda)\,d\lambda}{\int_{400nm}^{700nm} E_{solar}(\lambda)\,d\lambda \,/ \int_{0}^{\infty} E_{solar}(\lambda)\,d\lambda} = \frac{\eta_{PAR}^{ex}}{\eta_{PAR}} = \frac{0.338}{0.368} = 0.92\,. \qquad \text{(S41)}$$

Compared with eq.(S37b), this means that $r = 1.18$, small enough to change the efficiency of solar-photon to chemical energy conversion only quite minimally.

With $E_{\text{solar}} = h\,\nu = h\,c/\lambda = 200$ kJ/mol for $\lambda = 600$ nm, we obtain

$$\Delta_r G < 0.92\ E_{\text{photon}} = 0.92 \times 200 \text{ kJ/mol} = 184 \text{ kJ/mol}. \qquad \text{(S42)}$$

This is not enough to produce the ~450 kJ/mol needed to convert 2 H_2O, which is stable with 4 strong OH bonds, to O_2 with its relatively weak double bond.[8, 22] Therefore, absorption of multiple photons is required.

Nonequilibrium solar photon entropy. Based on Planck's book on The Theory of Heat Radiation (1959), Yourgrau and van der Merwe[17] presented an expression for the entropy of solar photons that are not in thermal equilibrium, as a function of light frequency ν:

$$S_{\text{photon}}(\nu) = (E_{\text{photon}}(\nu)/T_{\text{sun}})\ (kT_{\text{sun}}/h\nu)\ [-\log\xi\ + \exp(h\nu/kT_{\text{sun}})\ \log(1+\xi)] \qquad \text{(S43a)}$$

with $$\xi\ = 1/[\exp(h\nu/kT_{\text{sun}}) - 1]. \qquad \text{(S43b)}$$

In the frequency range of visible light, $h\nu/kT_{sun} \approx 3.6$ and thus $\exp(h\nu/kT_{sun}) >> 1$, so

$$\xi \approx \exp(-h\nu/kT_{sun}) << 1, \log\xi \approx -h\nu/kT_{sun},$$

and $$\log(1+\xi) \approx \xi \approx \exp(-h\nu/kT_{sun}).$$

Then eq.(43) simplifies to

$$S_{photon}(\nu) \approx (E_{photon}(\nu)/T_{sun})\ \ (1 + kT_{sun}/h\nu) \approx 1.28\ E_{photon}(\nu)/T_{sun}. \quad \text{(S44)}$$

Comparison with eq.(S34) shows that this corresponds to $r = 0.96$ in our previous analysis, which in eq.(S37) gives $\Delta_r G < 0.93\ E_{photon}$, very close to the result of the equilibrium theory. This is different from the maximum efficiency value of 0.88 given in ref.[17], probably because these authors considered the efficiency in terms of heat absorbed, i.e. enthalpy rather than free-energy change, and incorrectly assumed that the leaf's IR photon emission was tied to the energy absorption efficiency.

Photon absorption and chemical reactions. The absorption of four photons enables the endergonic reaction

$$4\ \text{P680} \rightarrow 4\ \text{P680}^{*} \qquad \Delta_r G^{o\prime} \approx +725\ \text{kJ/mol} > 0\ \ \text{(not at constant temperature).} \quad \text{(S45)}$$

All subsequent reactions are exergonic, starting with charge separation as pheophytin takes the "loosened" electron from P680*,

$$4\ \text{P680}^{*} + \text{Pheo} \rightarrow 4\ \text{P680}^{+\cdot} + \text{Pheo}^{-\cdot} \qquad \Delta_r G^{o\prime} \approx -60\ \text{kJ/mol} < 0 \quad \text{(S46)}$$

and subsequently $\text{P680}^{+\cdot}$ "charges up" Tyr_Z to the high-energy radical $\text{Tyr}_Z^{\cdot}$:

$$4\ \text{Tyr}_Z + 4\ \text{P680}^{+\cdot} \rightarrow 4\ \text{Tyr}_Z^{\cdot} + 4\ \text{P680} \qquad \Delta_r G^{o\prime} \approx -40\ \text{kJ/mol} < 0. \quad \text{(S47)}$$

In the water-splitting complex, the high-energy $\text{Tyr}_Z^{\cdot}$ achieves H transfer out of H_2O, and O_2 formation (by formation of 4 O-H bonds in 4 Tyr_Z), according to:

$$2\ H_2O + 4\ \text{Tyr}_Z^{\cdot} \rightarrow O_2 + 4\ \text{Tyr}_Z \qquad \Delta_r G^{o\prime} \approx -120\ \text{kJ/mol} < 0. \quad \text{(S48)}$$

The values for $\Delta_r G^{o\prime}$ are somewhat uncertain due to variations in the redox potentials of Tyr_Z and P680 reported in the literature.

Electron transport is made spontaneous by a *high*-energy acceptor. The “electron waterfall” concept, including the “downhill” portions of the Z-scheme, are easily taken to mean that electron transfer is thermodynamically favorable if it occurs from a high-energy donor to a low-energy acceptor. While this may seem intuitively appealing, the following analysis shows that it is wrong or confused on multiple accounts. Spontaneous electron transfer requires an acceptor that is sufficiently *high* in energy.

Attempts to write the electron-transfer from a donor D (e.g. Tyr_Z) to an acceptor A (e.g. P680), as seen in Figure S1 (lower left corner), as a simple chemical reaction $D \rightarrow A$, with or without electrons, are futile:

$Tyr_Z \rightarrow P680$ is not balanced.

$D \rightarrow A + e^-$ does not work because the electron did not get to the acceptor.

A more careful analysis shows that two half reactions must be involved, with (at least) *two* electron “donors” and *two* “acceptors”:

Donor 1 (oxidized) donates an electron: $\mathbf{D^{(1)}} \rightarrow A^{(1)} + e^-$ (S49)

(leaving behind a conjugate, reduced species $A^{(1)}$ that could act as an acceptor according to $A^{(1)} + e^- \rightarrow D^{(1)}$)

Acceptor 2 accepts an electron: $\mathbf{A^{(2)}} + e^- \rightarrow D^{(2)}$ (S50)

(producing a conjugate, oxidized species $D^{(2)}$ that could act as a donor according to $D^{(2)} \rightarrow A^{(2)} + e^-$).

Reactions (S50) and (S49) are a reduction and an oxidation, respectively. The overall redox reaction is

$$\mathbf{D^{(1)}} + \mathbf{A^{(2)}} \rightarrow A^{(1)} + D^{(2)}, \qquad \text{(S51)}$$

with the standard free energy of reaction

$$\begin{aligned}\Delta_r G^{o'} &= \text{“products – reactants”} \\ &= G^{o'}_{A(1)} + G^{o'}_{D(2)} - (\boldsymbol{G^{o'}_{A(2)}} + G^{o'}_{D(1)}). \qquad \text{(S52)}\end{aligned}$$

Clearly, the donor $D^{(1)}$ passes an electron to the acceptor $A^{(2)}$, so $A^{(2)}$ is the acceptor that actually accepts an electron in this reaction. The $- G^{o'}_{A(2)}$ term in eq.(S52) shows that $\Delta_r G^{o'}$ becomes

more negative and the reaction gives off more free energy if the acceptor receiving the electron is of *higher* free energy. The prime example is $O_2 + 4\ H^+$ in respiration.[8] While it is true that the reaction of a donor $\mathbf{D^{(1)}}$ is energetically more favorable the lower the energy of its *own* conjugate acceptor $\mathbf{A^{(1)}}$, that acceptor does not accept an electron in the reaction. The acceptor that does ($\mathbf{A^{(2)}}$) is always the acceptor of a *different* redox pair (the other half reaction). That acceptor is a reactant, see eq.(S51), so the higher its energy, the more energy is released by the reaction.

Pseudo energy levels of half reactions. Instead of overall redox reactions like eq.(S51), the corresponding half reactions as in eq.(S49) and (S50) are emphasized in the Z-scheme and other traditional representations of photosynthesis and oxidative phosphorylation. In electron transport chains with many intermediates, this has some merit in terms of simplicity (even though sequences of RETAR diagrams like the EZ-scheme, see Figures 1-5 and S10-12, can convey the same information more clearly). One can express the free energy of half reactions (S50) and the reverse of (S49) [i.e. both as reductions], $\Delta_{hr}G^{o'(2)}$ and $\Delta_{hr}G^{o'(1)}$, respectively, in terms of the free energies of the donors $G^{o'}{}_{D(1)}$ and $G^{o'}{}_{D(2)}$ and the acceptors $G^{o'}{}_{A(1)}$ and $G^{o'}{}_{A(2)}$:

$$\Delta_{hr}G^{o'(1)} = G^{o'}{}_{D(1)} - G^{o'}{}_{A(1)} \qquad \text{(S53a)}$$

$$\Delta_{hr}G^{o'(2)} = G^{o'}{}_{D(2)} - G^{o'}{}_{A(2)}. \qquad \text{(S53b)}$$

The standard free-energy change in the combined redox reaction is related according to

$$\begin{aligned} \Delta_r G^{o'} &= G^{o'}{}_{A(1)} + G^{o'}{}_{D(2)} - (G^{o'}{}_{A(2)} + G^{o'}{}_{D(1)}) \\ &= (G^{o'}{}_{D(2)} - G^{o'}{}_{A(2)}) - (G^{o'}{}_{D(1)} - G^{o'}{}_{A(1)}) \\ &= \Delta_{hr}G^{o'(2)} - \Delta_{hr}G^{o'(1)}. \end{aligned} \qquad \text{(S54)}$$

In Figure S3a, the free-energy levels of reactants and products are shown schematically. In Figure S3b, $\Delta_{hr}G^{o'(1)}$ and $\Delta_{hr}G^{o'(2)}$ are sketched as pseudo energy levels, like those in the Z-scheme of photosynthesis, where in addition the free-energy differences are shifted and then encrypted as standard reduction potentials in volts. Since the difference between the energy levels in both figures is $\Delta_r G^{o'}$, they look deceptively similar. However, the meaning of the energy levels is very different: each of the levels in Figure S3b mixes contributions from reactants and products.

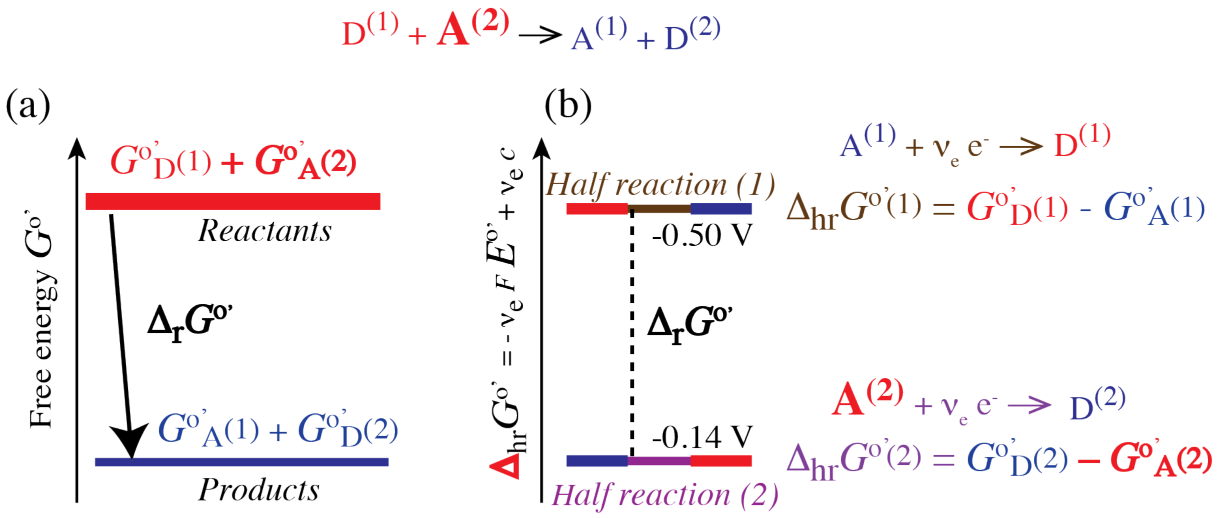

Figure S3. (a) Standard free-energy levels $G^{o'}$ of reactants and products in a redox reaction shown schematically. (b) Half-reaction pseudo energy levels $\Delta_{hr}G^{o'(1)}$ and $\Delta_{hr}G^{o'(2)}$ as in the Z-scheme of photosynthesis.

Equations (S52) – (S54) are based on meaningful, individual standard free energies G^o referenced to the free atoms at zero free energy. Values of G^o for important molecules, many metals, and metal ions in aqueous solution are listed in tables in the Supporting Information of references [23] and [8]. The conventional analogue of G^o, the free energy of formation, $\Delta_f G^o$, is referenced to the standard hydrogen electrode and its half reaction, analyzed below, and therefore reflects not only the energetics of the donor or acceptor species but also the bond, ionization, and hydration energies of hydrogen.

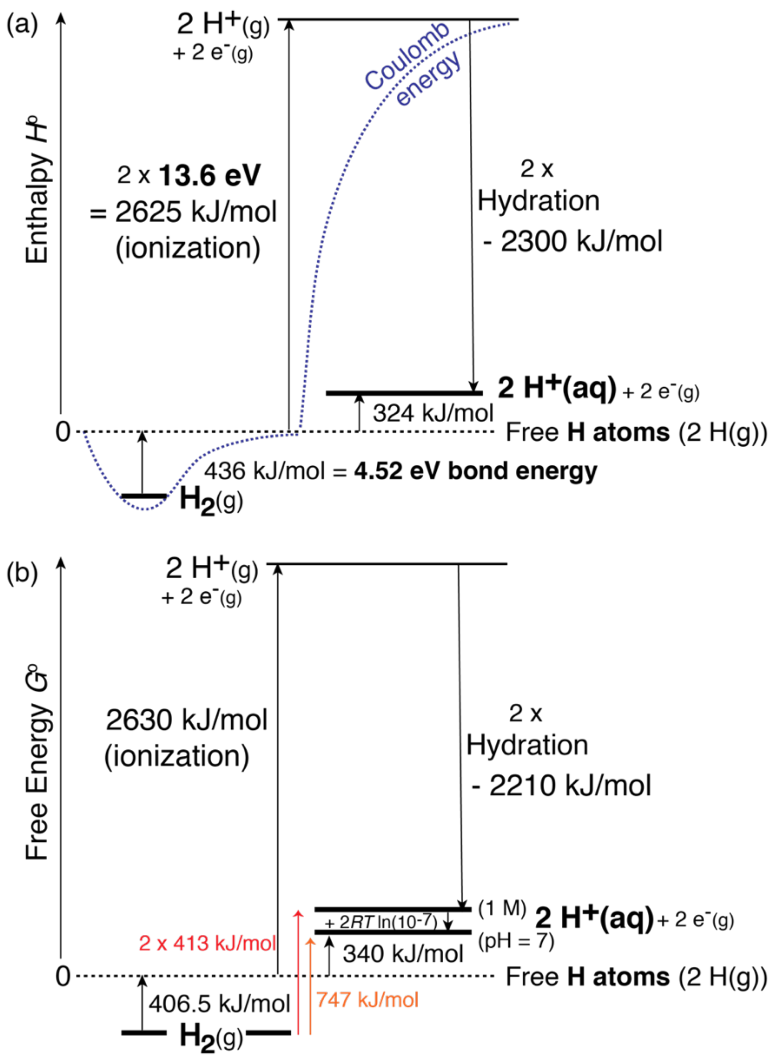

Figure S4. Explanation of the energy difference between H_2(g) and $2H^+$(g) (a) in terms of familiar energy (enthalpy) differences and (b) in the corresponding free-energy diagram, with moderate entropic contributions added to the enthalpy differences from (a).

Understanding and calculating $\Delta_{hr}G^{o'}$ values. An 'energy level' in the Z-scheme can represent the free-energy *difference* $\Delta_{hr}G^{o'}$ between reduced and oxidized species in the half reaction in question. The same energy difference is represented by a curved, mostly vertical arrow in a RETAR diagram. How these energetics can be understood in intuitive terms is demonstrated here for the hydrogen half reaction

$$2\ H^+(aq) + 2\ e^- \rightarrow H_2(g) \qquad \Delta_{hr}G^o = 2(-413\ \text{kJ/mol}),\ E^o = 0\ \text{V} \qquad \text{(S55a)}$$

$$\Delta_{hr}G^{o'} = -747\ \text{kJ/mol},\ E^{o'} = -0.41\ \text{V} \qquad \text{(S55b)}$$

whose energy values, partly familiar from introductory physical chemistry, are summarized in Figure S4.

In Figure S4a, enthalpy changes are shown: To convert H_2 to two free H atoms, the bond energy of 436 kJ/mol = 4.52 eV has to be put in. Ionization of two H atoms in the gas phase (familiar from basic quantum mechanics) requires 2 × 13.6 eV = 2625 kJ/mol, which is partially offset by the hydration or solvation energy of 2 × 1150 kJ/mol (according to Tissandier et al., J. Phys. Chem. A 102, 7787, 1998) as 2 H^+(aq) are formed. The energy of the free electrons is ignored (considered to be zero) since electrons are only intermediates in the overall redox reaction; they do not appear among reactants or products and therefore do not affect the overall energetics of the full redox reaction. In Figure S4b, the corresponding, quite similar *free-energy* changes are shown. The free-energy difference between H_2 and H^+(aq) at 1 mol/L concentration is -(406.5 + 2630) kJ/mol + 2210 kJ/mol = 2(-413 kJ/mol). At pH 7 and T = 300 K, this is changed by – 2 $RT \ln(10^{-7})$ = 80 kJ/mol and we obtain $\Delta_{hr}G^{o'}$ = -747 kJ/mol, see Figure S4b. The relation of the free energy of this half reaction at 1 mol/L and the absolute standard reduction potential of the standard hydrogen electrode is discussed further below.

Free energies of half reactions and standard reduction potentials: Overview. Free energies of half reactions in kJ/mol are rarely discussed directly in traditional descriptions of biochemical redox reactions. Instead, standard reduction potentials $E^{o'}$, in volts, are widely used as stand-ins, even though energies in volt will appear as arcane to the uninitiated. In the traditional framework, the formal relation would be

$$\Delta_{hr,t}G^{o'} = - \nu_e F E^{o'} \qquad \text{(S56)}$$

where F is Faraday's constant and ν_e is the stoichiometric coefficient of the electrons transferred; in eq.(S55), ν_e = 2. The derivation of this relation is outlined below. Readers interested simply in understanding photosynthesis and not its historical cloaking in standard reduction potentials can skip the following theory down to at least eq.(S98). We want to emphasize here that standard reduction potentials in volts are not necessary for understanding photosynthesis; the EZ-scheme developed in this paper relies simply on free-energy values in kJ/mol. However, those who need to understand the problems with the use of standard reduction potentials in photosynthesis may want to read on. The relations given in this section enable quantitative conversion between the EZ-scheme with energies in kJ/mol and the Z-scheme with its pseudo energy levels in volts.

The meaningful free energy of half-reaction, $\Delta_{hr}G^{o'}$ (without the subscript $_t$) as introduced above, e.g. in eq.(S53), relates to the *absolute* standard reduction potential $E_a^{o'}$ according to

$$\Delta_{hr}G^{o'} = - \nu_e F E_a^{o'} \qquad \text{(S57)}$$

analogous to eq.(S56) and also derived below. Redox potentials in V correspond directly to free-energy differences in eV, most clearly if one acknowledges that $F = e$ since 1 mol electrons = 6.022×10^{23} electrons (J. Chem. Educ. 97, 597 (2020)). The half reaction in the standard hydrogen electrode analyzed above can provide the relation between the traditional and absolute free energy of half reaction, $\Delta_{hr,t}G^{o'}$ and $\Delta_{hr}G^{o'}$, respectively. The conventional standard reduction potential of the hydrogen half reaction in eq.(S55) is $E^o = 0$ V, while the absolute value according to Figure S4b is

$$E_a^o = - \Delta_{hr}G^{o'}/(\nu_e F) = -2(-413 \text{ kJ/mol})/(2F) = 4.28 \text{ V}. \qquad \text{(S58)}$$

Thus, absolute and conventional values are related according to

$$E_a^{o'} = E^{o'} + 4.28 \text{ V}. \qquad \text{(S59)}$$

This means that

$$E_a^{o'(2)} - E_a^{o'(1)} = E^{o'(2)} - E^{o'(1)}. \qquad \text{(S60)}$$

Combining the two equations (S57) and (S59), we obtain

$$\Delta_{hr}G^{o'} = - \nu_e F (E^{o'} + 4.28 \text{ V}) = - \nu_e F E^{o'} - \nu_e\, 413 \text{ kJ/mol}. \qquad \text{(S61)}$$

With eq.(S56), this also means that the traditional value

$$\Delta_{hr,t}G^{o'} = \Delta_{hr}G^{o'} + \nu_e\, 413 \text{ kJ/mol} \qquad \text{(S62)}$$

has a confounding shift by ν_e 413 kJ/mol, and equivalently

$$\Delta_{hr,t}G^{o'} = G^{o'}{}_D - G^{o'}{}_A + \nu_e\, 413 \text{ kJ/mol} \qquad \text{(S63)}$$

where the last equality used eq.(S53). This means that a conventional or traditional redox-potential energy $\Delta_{hr,t}G^{o'}$ is not just the difference between the free energies of reduced and oxidized species. For instance, if $E^{o'} = 0$ V, from eq.(S56) one easily calculates $\Delta_{hr,t}G^{o'} = 0$ kJ/mol, but according to

eq.(S63) this does not mean that reduced and oxidized species in the half reaction have the same free energy at biological standard conditions. In reality, their actual free energies differ by $\Delta_{hr}G^{o'} = -\nu_e$ 413 kJ/mol. Since its value has been routinely encrypted in volts, this unphysical shift of $\Delta_{hr,t}G^{o'}$ has remained unaddressed in conventional descriptions of bioenergetics.

Applying eq.(S62) back to the hydrogen half reaction in eq.(S55) as an example, we obtain the values of $\Delta_{hr}G^{o}$ and $\Delta_{hr}G^{o'}$ shown in Figure S4b. From eqs.(S62) and (S54), we find that the *differences* between traditional free-energy differences and meaningful free-energy differences of two half reactions are the same:

$$\Delta_{hr,t}G^{o'(2)} - \Delta_{hr,t}G^{o'(1)} = \Delta_{hr}G^{o'(2)} - \Delta_{hr}G^{o'(1)} = \Delta_{r}G^{o'} = - \nu_e F \Delta E^{o'}. \qquad \text{(S64)}$$

When reduction potentials are actual voltages. In the following, we outline the derivation of the relation between free-energy change in a redox reaction and the cell potential (electromotive force), which underlies eq.(S56). It will become apparent that the presence of a physical voltage in redox systems is linked to the presence of conductive electrodes.

In an electrochemical reaction at constant T & P,

$$\mathrm{d}G = \Sigma_i \, \tilde{\mu}_i \, \mathrm{d}n_i \qquad \text{(S65)}$$

with electrochemical potentials $\tilde{\mu}_i$ (chemical potentials with an electric potential term added) and the sum over all chemical species and the electrons in different environments, see Mortimer, Physical Chemistry, 2008. The changes in the amounts of reactants, products, and electrons are linked to the extent of reaction ξ (in moles) through the unitless stoichiometric coefficients $\tilde{\nu}_i$ (with a minus sign for any reactant, since its amount decreases):

$$\mathrm{d}n_i = \tilde{\nu}_i \, \mathrm{d}\xi. \qquad \text{(S66)}$$

Then

$$\mathrm{d}G = \Sigma_i \, \tilde{\mu}_i \, \mathrm{d}n_i = \Sigma_i \, \tilde{\mu}_i \, \tilde{\nu}_i \, \mathrm{d}\xi. \qquad \text{(S67)}$$

This becomes $$\mathrm{d}G/\mathrm{d}\xi = \Sigma_i \, \tilde{\mu}_i \, \tilde{\nu}_i. \qquad \text{(S68)}$$

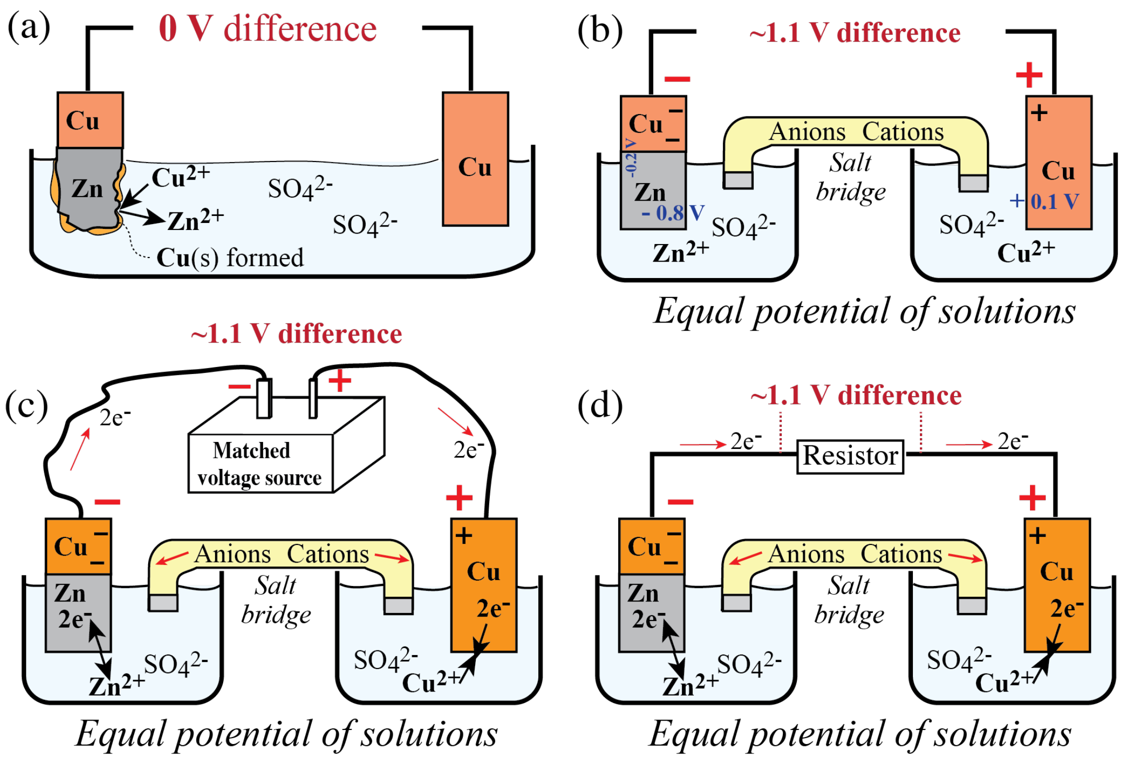

Figure S5. Reactions of metallic zinc and aqueous Cu^{2+} ions (a) without a voltage and (b-d) with generation of a voltage. (Note that the initial presence of Zn^{2+} ions is optional and that the copper electrode could in principle be replaced with a platinum electrode.) (a) Direct reaction of Zn(s) and Cu^{2+}(aq) ions, producing Cu(s) and Zn^{2+}(aq) and releasing 218 kJ/mol of heat; this reaction is utilized in heat packs. The redox reaction produces no electric potential difference under these conditions, as discussed in the text further below. (b-d): Electrochemical Daniell-type combinations of half-cells with actual voltages arising from electron accumulation and/or depletion in the electrodes [24] in electrochemical equilibrium, reflecting the simultaneous presence of reduced and oxidized species (e.g. Zn^{2+} and $Zn^{(0)}$) in each half cell. Electrical energy of 213 kJ/mol and heat of 5 kJ/mol is released under ideal conditions. The zinc electrode is connected to a piece of copper in order to avoid the effect of a Volta or Galvani contact potential difference between the electrodes. Ion concentrations are assumed to be 1 mol/L. (b) Open cell with negative charges on the zinc electrode due to putative dissolution of some metallic zinc as Zn^{2+}(aq) and positive charges on the copper electrode due to reduction of some Cu^{2+}(aq) to metallic copper.[24] Estimated interfacial potential drops are indicated in blue. (c) Reversible cell with a matched applied voltage that is nearly equal in magnitude and opposite in sign to the cell voltage. (d) Set-up analogous to c) with the nearly matched external voltage generated by a resistor of large resistance. In b) – d), the cell voltage is $E_{cell} = E_{cell}^{o'} - RT/(\nu_e F) \ln Q'$.

The electrochemical potentials for reactants and products without a liquid junction potential between half cells are the usual chemical potentials, for instance in terms of molarities [i] in M = mol/L:

$$\tilde{\mu}_i = \mu_i^{ref} + RT \ln([i]/M) \qquad (S69)$$

Protons are given special treatment in biochemistry, with

$$\tilde{\mu}_{H+} = \mu_{H+}^{ref} + RT \ln([H^+]/(10^{-7} M)). \qquad (S70)$$

For "free" electrons in a conductive electrode at an electrical potential $\phi_{\pm}$,

$$\tilde{\mu}_{ele,\pm} = \mu_{ele}^{met} - F\,\phi_{\pm} \qquad (S71)$$

with the minus sign in front of $F\,\phi$ from the negative charge of electrons, see Mortimer's book. The electron-deficient positive electrode is at the potential $\phi_+ > \phi_-$, which is greater than the potential ϕ_- of the negative electrode since electric potential is higher near positive charges. The potential difference is generated by difference in electric charge due to the electron deficiency and excess on the two conductive electrodes. In the absence of such electrodes in biochemical electron transport chains, there is no such charge and resulting potential difference.

To avoid complications, the potential difference between electrons in the *same* metal (or graphite) is considered in the following. For instance, one can connect a zinc electrode opposite a copper electrode to a piece of copper (see Figure S5). Then the equation applies to the electrons in copper at both electrodes.

The condition for equilibrium or for a reversible cell is

$$dG = \delta w_{ele}. \qquad (S72)$$

In the following, we analyze two equilibrium situations, the open and the reversible electrochemical cell.

Open electrochemical cell. An electrically open electrochemical cell as shown in Figure S5b (note that it is still a closed thermodynamic system) does not drive any electrons and therefore does no work

$$dG = \delta w_{ele} = 0 \quad \text{and} \quad dG/d\xi = 0. \qquad (S73)$$

Under these conditions, eq.(S73) can be evaluated using the usual quantities in chemical thermodynamics:

$$0 = \mathrm{d}G/\mathrm{d}\xi = \Sigma_i \ \tilde{\mu}_i \ \tilde{\nu}_i = \ \Delta_r G^{o'} + RT \ln Q' - \nu_e \, F \, \Delta\phi \qquad \text{(S74)}$$

with $\Delta_r G^{o'} = \Sigma_i \ \mu_i^{ref} \tilde{\nu}_i$ as usual and the μ_{ele}^{met} terms canceling. Since for the reaction written in the spontaneous direction, electrons are products on the negative electrode, $\Delta\phi = \phi_{products} - \phi_{reactants} = \phi_- - \phi_+ < 0$. We also have the familiar, unitless reaction quotient in equilibrium, Q', which can be formally written as the product

$$Q' = \prod_{i=1}^{n_{species}} ([i]/M)^{\tilde{\nu}_i} \qquad \text{(S75)}$$

(with special ([H^+]/10^{-7} M) in biochemistry). We note that dG/dξ is often written as $\Delta_r G$, but this violates the fundamental definition of Δ in thermodynamics, first introduced in $\Delta U = w + q$. Notably, the units do not match. Correctly, $\Delta G = G_{final} - G_{initial}$ has units of kJ, while dG/dξ has units of kJ/mol.

Now we can solve eq.(S74) for the potential difference or voltage between the copper ends of the two electrodes that has been generated by the electrochemical reaction in the cell:

$$-\Delta\phi = \Delta_r G^{o'}/(-\nu_e F) + RT/(-\nu_e F) \ln Q'. \qquad \text{(S76)}$$

This is the electromotive force E_{cell} (a good discussion can be found in David Griffiths' Introduction to Electrodynamics) in volts, generated by the electrochemical cell according to Mortimer:

$$E_{cell} = -\Delta\phi = \Delta_r G^{o'}/(-\nu_e F) - RT/(\nu_e F) \ln Q'. \qquad \text{(S77)}$$

Under standard conditions, by definition [i] = 1 M except [H^+] = 10^{-7} M, which gives $Q' = 1$ and $\ln Q' = 0$ according to eq.(S75), so we obtain the standard cell voltage

$$E_{cell}^{o'} = \Delta_r G^{o'}/(-\nu_e F) \qquad \text{(S78)}$$

marked by the standard-condition superscript ^{o}. This converts eq.(S77) to the famous Nernst equation:

$$E_{cell} = E_{cell}^{o'} - RT/(\nu_e F) \ln Q'. \qquad \text{(S79)}$$

Reversible electrochemical cell. Next we consider an electrochemical system consisting of two half cells, as usual with a salt bridge or other mechanism to avoid a liquid junction potential, and with small, nearly negligible current flow due to a matched applied counter-voltage $\Delta\phi_{applied} \approx \Delta\phi$, see Figure S5c. This situation is conceptually analogous to the well-known reversible expansion of a gas, where the applied pressure P_{ex} essentially matches the system pressure P, i.e., $P_{ex} \approx P$. Such a process is reversible in the strict thermodynamics sense: A slight variation of the counter-voltage reverses the current flow. In other words, the cell is at the tipping point between a galvanic/voltaic cell and an electrolytic cell.

Here, the "amount" (i.e., the number) n_{ele} of electrons on an electrode can change in two ways:

(i) by the chemical reaction inside the cell; this is still included in $(\Sigma_i\ \tilde{\mu}_i\ \tilde{\nu}_i)\ d\xi$;

(ii) by a current dQ_{ele}/dt outside the cell, where $dn_{ele,curr} = \pm\ dQ_{ele}/F$.

(Since it can be shown that $F = e$, see J. Chem. Educ. 97, 597, 2020, readers with a physics background may mentally replace the Faraday constant F with the elementary charge e.)

Both of these contributions to dn_{ele} multiply $\tilde{\mu}_{ele,\pm} = \mu_{ele}^{met} - F\ \phi_{\pm}$. Thus, showing only the second contribution explicitly at this point, we can write

$$dG = \Sigma_i\ \tilde{\mu}_i\ dn_i = (\Sigma_i\ \tilde{\mu}_i\ \tilde{\nu}_i)\ d\xi - \phi_-\ F\ dQ_{ele}/F - \phi_+\ F\ (-dQ_{ele}/F) \qquad (S80)$$

$$= (\Sigma_i\ \tilde{\mu}_i\ \tilde{\nu}_i)\ d\xi - \Delta\phi\ dQ_{ele}. \qquad (S81)$$

We see that dQ_{ele} is the charge loss or gain of the negative electrode into or out of the external circuit. If electrons flow spontaneously from the negative to the positive electrode, $dQ_{ele} < 0$, while in electrolysis, electrons are 'pumped' from the positive to the negative electrode, and $dQ_{ele} > 0$; note that in either case, the negative electrode is physically the same, e.g. the zinc electrode in Figure S5c. Since electrical work is the transported charge dQ_{ele} times the electric potential drop $\Delta\phi$ through which the charge moves, we have

$$\delta w_{ele} = -\ \Delta\phi\ dQ_{ele}. \qquad (S82)$$

The minus sign can be attributed to the physical-chemistry sign convention for work, as in the familiar $-\ P\ dV$. Note that the applied voltage $\Delta\phi$ is of significant magnitude, $|\Delta\phi| = E_{cell}$, rather than the negligible difference $|\Delta\phi\ | - |E_{cell}|$: If one measures the voltage between the electrodes of a battery or galvanic cell connected to a charger when the battery or galvanic cell is fully charged, one will find a significant voltage of magnitude $|\Delta\phi| = E_{cell}$. Thus, even though the galvanic and the applied voltage almost match, the electrons still do the work given in eq.(S82), just as an expanding gas lifting a piston does work $-\ P\ dV$ with the full pressure P even when the

internal gas pressure and the external pressure exerted by the piston are almost the same in a reversible expansion. Note also that the change in the sign of electrical work from a spontaneous galvanic to a driven electrolytic cell does not come from a sign change in $\Delta\phi$ but rather one in dQ_{ele}, just like the sign change from expansion to compression work does not come from a sign change in pressure P but rather in volume change dV. A close mechanical analogue of an electrochemical cell, which is of constant total charge, is a constant-volume container consisting of two gas-filled "half cells" at different pressures and variable volume due to a piston separating them. Here, $\delta w_{PV} = - \Delta P\ dV_{hiP}$, in quite close analogy to eq.(S82).

Combining these equalities eqs.(S80) – (S82), we obtain

$$- \Delta\phi\ dQ_{ele} = \delta w_{ele} = dG = (\Sigma_i\ \tilde{\mu}_i\ \tilde{\nu}_i)\ d\xi - \Delta\phi\ dQ_{ele}, \qquad \text{(S83)}$$

which simplifies to

$$0 = dG/d\xi = \Sigma_i\ \tilde{\mu}_i\ \tilde{\nu}_i\ . \qquad \text{(S84)}$$

This is the same as the first two equalities in eq.(S74) for the open cell, so

$$0 = \Sigma_i\ \tilde{\mu}_i\ \tilde{\nu}_i = \Delta_r G^{o'} + RT\ \ln Q' - \nu_e\ F\ \Delta\phi. \qquad \text{(S85)}$$

Then the Nernst equation results again:

$$-\Delta\phi = E_{cell} = E_{cell}^{o'} - RT/(\nu_e F)\ \ln Q'. \qquad \text{(S86)}$$

Free energies of half reactions and standard reduction potentials: Derivation. Equation (S78) or (S56) for an (electrically) open or reversible electrochemical cell with electrodes and no liquid junction potential is equivalent to

$$\Delta_r G^{o'} = -\nu_e F\ E_{cell}^{o'}. \qquad \text{(S87)}$$

Combining this with eq.(S54), $\Delta_r G^{o'} = \Delta_{hr} G^{o'(2)} - \Delta_{hr} G^{o'(1)}$, we get

$$-\nu_e F\ E_{cell}^{o'} = \Delta_r G^{o'} = \Delta_{hr} G^{o'(2)} - \Delta_{hr} G^{o'(1)}, \qquad \text{(S88)}$$

which yields

$$E_{cell}^{o'} = \Delta_{hr}G^{o'(2)}/(-\nu_e F) - \Delta_{hr}G^{o'(1)}/(-\nu_e F). \quad (S89)$$

If we define, equivalent to eq.(S56), an absolute standard reduction potential as

$$E_a^{o'} = \Delta_{hr}G^{o'}/(-\nu_e F) \quad (S90)$$

Eq.(S89) becomes

$$E_{cell}^{o'} = E_a^{o'(2)} - E_a^{o'(1)}. \quad (S91)$$

According to eq.(S60), this is also the difference between conventional standard reduction potentials relative to the hydrogen electrode, so

$$E_{cell}^{o'} = E_a^{o'(2)} - E_a^{o'(1)} = E^{o'(2)} - E^{o'(1)} = \Delta E^{o'}. \quad (S92)$$

In the conventional framework, one similarly obtains $\Delta_r G^{o'} = \Delta_{hr,t}G^{o'(2)} - \Delta_{hr,t}G^{o'(1)}$ according to eq.(S64) and can write

$$-\nu_e F\,\Delta E^{o'} = \Delta_r G^{o'} = \Delta_{hr,t}G^{o'(2)} - \Delta_{hr,t}G^{o'(1)} \quad (S93a)$$

$$\Delta E^{o'} = \Delta_{hr,t}G^{o'(2)}/(-\nu_e F) - \Delta_{hr,t}G^{o'(1)}/(-\nu_e F) \quad (S93b)$$

so one can define a standard reduction potential as

$$E^{o'} = \Delta_{hr,t}G^{o'}/(-\nu_e F) \quad (S94)$$

which yields eq.(S56).

A voltage only under the right conditions. The use of redox potentials in volts in the Z-scheme of photosynthesis suggests that the electron transport in the photosystems is driven by voltages. This is not the case. Here we show that a redox reaction produces a voltage $\Delta E^{o'}$ only under certain conditions, while its free-energy change $\Delta_r G^{o'}$ applies very generally. In biochemistry, the left-hand side of $\Delta E^{o'} = \Delta_r G^{o'}/(-\nu_e F)$ is often only a formal stand-in for the free-energy difference $\Delta_r G^{o'}$.

As a simple example, we consider the well-known redox reaction

$$Zn(s) + Cu^{2+}(aq) \rightarrow Zn^{2+}(aq) + Cu(s) \qquad \Delta_r G^o = -213 \text{ kJ/mol} \quad (S95)$$

and its half reactions

$$Zn(s) \rightarrow Zn^{2+}(aq) + 2\ e^- \qquad E^o = -0.76\ V\ \text{(for the reduction)} \qquad (S96)$$

$$Cu^{2+}(aq) + 2\ e^- \rightarrow Cu(s) \qquad E^o = +0.34\ V. \qquad (S97)$$

If $Cu^{2+}(aq)$ reacts directly with a rod of zinc metal, i.e., there is no separation into half cells, as shown in Figure S5a, no electrical work is done, so $w_{ele} = 0$ and $\Delta_r H^o = -218$ kJ/mol is released as heat. Electron transfer from Zn to Cu does occur here but it is not associated with a voltage, since $V_{ele} = -\ w_{ele}/(2F) = 0 \neq (0.34\ V - (-0.76\ V)$. A voltage that is equal to the differences in $E^{o\prime}$ arises specifically from the local accumulation or depletion of electrons in a conductive electrode, driven by a pair of half reactions whose solutions are in ion-exchange contact (i.e., without a liquid junction potential).[24] These conditions do not apply during electron transport reactions in the thylakoid membrane.

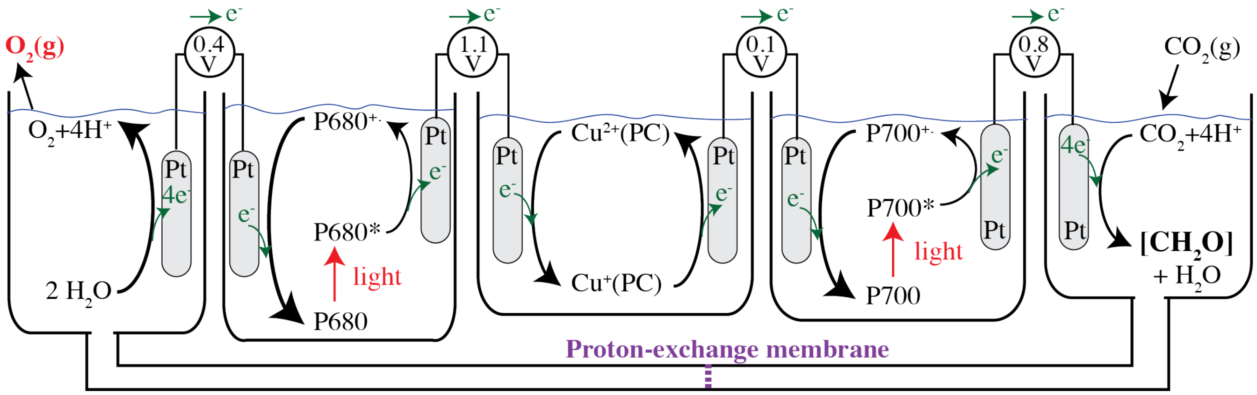

Figure S6. Cartoon of a series of hypothetical galvanic cells with inert platinum electrodes and crucial photosynthetic redox reactions (suggested by Stefan Stoll). The numerous redox cycles left out could be added in by analogy to the plastocyanin (PC) cycle shown in the center of the figure. The vertical range of a curved black arrow represents the free-energy difference $\Delta_{hr}G^{o\prime}/\nu_e$ or absolute standard reduction potential $E_a^{o\prime}$ of the half reaction. A close formal analogy to the EZ-scheme in Figure 4 can be observed. Under light irradiation and with sufficiently fast kinetics, the hypothetical set-up would continuously produce carbohydrate $[CH_2O]$, high-energy O_2, and some electrical energy. Additionally, if ATP synthase was inserted in the proton-exchange membrane (purple, center bottom), which only passes $H^+(aq)$ and H_2O, the chemical energy of the generated H^+ concentration gradient could be used to convert ADP + P_i to ATP + H_2O. Due to the presence of conducting electrodes, and coexistence of conjugate redox species in equilibrium, redox potential differences would be observed as voltages (electric potential differences between electrodes due to their slight relative electron excess or deficiency) here. In the thylakoid membrane, these conditions are not fulfilled and voltages directly corresponding to redox potential differences do not exist.

The difference in electrical work produced by the Zn(s) + Cu^{2+}(aq) redox reaction in one cell (Figure S5a) vs. reversibly in two half-cells with electrodes (Figure S5b), 0 vs. -213 kJ/mol, respectively, is an example of the path dependence of work emphasized in introductory thermodynamics. The Gibbs free energy, by contrast, is a state function whose change is path independent, so $\Delta_r G^{o'}$ applies, at least approximately, under a wide range of conditions. Analogously, a biochemical half reaction *in vitro* with an electrode inserted may produce a voltage relative to a reference electrode or other half reaction (see Figure S6) but that does not mean that a voltage exists *in vivo*.

Traditional Gibbs free energies of half reaction are meaningless. The traditional free-energy difference $\Delta_{hr,t}G^{o'}$ associated with a traditional standard reduction potential according to eq.(S56) is not the free energy of the given half reaction, but involves the ionization energy of atomic hydrogen and the bond energy of H_2. For instance, $\Delta_{hr,t}G^o$ for the half reaction Fe^{3+}(aq) + e^- → Fe^{2+}(aq) is really the standard free-energy change of the following reaction:

$$Fe^{3+}(aq) + \tfrac{1}{2} H_2 \rightarrow Fe^{2+}(aq) + H^+(aq) \qquad \text{(S98a)}$$

$$\Delta_{hr,t}G^o = - F \times 0.77\ \text{V} = -74.3\ \text{kJ/mol}. \qquad \text{(S98b)}$$

While energy differences ΔG are usually independent of the choice of zero point of energy, this is not true for these energy differences $\Delta_{hr,t}G^o$. According to eqs.(S98a) and (S62), they are different from the true differences $\Delta_{hr}G^{o'}$ between the actual free energies of reduced and oxidized species (Fe^{3+}(aq) and Fe^{2+}(aq) in the example) as introduced in eq.(S53) by - ν_e 413 kJ/mol, since H_2 and H^+(aq) appear in the reaction of eq.(S98a). Only the *difference* of these free-energy differences for the two half reactions combining is meaningful, since according to eq.(S64) it equals $\Delta_r G^{o'} = - \nu_e F \Delta E_o'$ of the overall redox reaction. Here the contributions from H^+ and H_2 to each of the two $\Delta_{hr,t}G^o$ values have canceled.

Reduction potentials as formal stand-ins for reaction free energy. Why have non-existent reduction potentials and their associated meaningless energies $\Delta_{hr,t}G^o$ nevertheless been useful in traditional bioenergetics? It is the *difference* of free-energy differences or standard reduction potentials that is meaningful,[8] being equal to the standard free-energy change in the overall redox reaction, according to eq.(S93a):

$$- \nu_e F \Delta E^{o\prime} = \Delta\Delta_{hr,t}G^{o\prime} = \Delta\Delta_{hr}G^{o\prime} = \Delta_r G^{o\prime} \qquad \text{(S93a')}$$

Thus, from the difference $\Delta E^{o'}$ between the standard reduction potentials "of Pheo" and "of Q_A", which are $E^o_{Pheo, Pheo^{-\cdot}}$ = -0.505 V and $E^o_{QA, QA^{-\cdot}}$ = -0.144 V, respectively, we can predict that the full redox reaction $Pheo^{-\cdot} + Q_A \rightarrow Pheo + Q_A^{-\cdot}$ has

$$\Delta_r G^{o'} = - F\,[-0.144\ V - (-0.505\ V)] = -35\ kJ/mol \ll 0.$$

In particular, this shows that $\Delta_r G^{o'}$ is significantly negative, which guarantees that the redox reaction is spontaneous. This formal relation (S93a) makes empirical standard reduction potentials useful, but does not prove in any way that corresponding voltages exist and drive electrons in electron transport chains.

Individual standard Gibbs free energies of ions from their free energies of formation. The absolute free energies of reduced and oxidized species in the EZ-scheme can be obtained from tabulated traditional standard molar Gibbs energies of formation.[25] As a specific example, we consider the reduction of ferredoxin:

$$Fe^{3+}(ferredox) + e^- \rightarrow Fe^{2+}(ferredox) \quad E^{o'} = -0.42\ V\ (\Delta_r G^{o'} = 40.5\ kJ/mol). \quad (S99)$$

According to data listed by Alberty,[25] the free energy of formation of ferredoxin (reduced) or Fe^{2+}(ferredox) is +38.1 kJ/mol (independent of [H^+] and [Mg^{2+}]):

$$Fe(s) + ferredox + 2\ H^+(aq) \rightarrow Fe^{2+}(ferredox) + H_2 \quad (S100)$$
$$\Delta_f G^{o''} = 38.1\ kJ/mol + c$$

compared with

$$Fe(s) + ferredox + 2\ H^+(aq) \rightarrow Fe^{2+}(aq) + ferredox + H_2 \quad (S101)$$
$$\Delta_f G^{o''} = -78.9\ kJ/mol + c.$$

Subtracting the two equations, we obtain

$$Fe^{2+}(aq) + ferredox \rightarrow Fe^{2+}(ferredox) \qquad \Delta_r G^{o''} = +117\ kJ/mol. \quad (S102)$$

Thus, Fe^{2+}(ferredox) is 117 kJ/mol higher in free energy than separate ferredoxin and Fe^{2+}(aq), whose individual free energy is 368 kJ/mol (see Table S1 in ref.[8]), and the absolute standard free energy of Fe^{2+}(ferredox) is

$$G^{o''}_{Fe2+(ferredox)} = 368\ kJ/mol + 117\ kJ/mol = 485\ kJ/mol \quad (S103)$$

The free energy of formation of ferredoxin (oxidized) or Fe^{3+}(ferredox) is -0.8 kJ/mol:[25]

$$Fe(s) + ferredox + 3\ H^+(aq) \rightarrow Fe^{3+}(ferredox) + 1.5\ H_2$$
$$\Delta_f G^{o''} = -0.8\ kJ/mol + c' \quad (S104)$$

compared with

$$Fe(s) + ferredox + 3\ H^+(aq) \rightarrow\ Fe^{3+}(aq) + ferredox + 1.5\ H_2 \qquad (S105)$$

$$\Delta_f G^{o"} = -4.7\ kJ/mol + c'$$

Subtracting the two equations, we obtain

$$Fe^{3+}(aq) + ferredox \rightarrow Fe^{3+}(ferredox) \qquad \Delta_r G^{o"} = +3.9\ kJ/mol \qquad (S106)$$

Thus, Fe^{3+}(ferredox) is 3.9 kJ/mol higher in energy than separate ferredoxin and Fe^{3+}(aq), whose individual free energy is 856 kJ/mol (see Table S1 in ref.[8]), and therefore

$$G^{o"}_{Fe3+(ferredox)} = 856\ kJ/mol + 3.9\ \ kJ/mol = 859.9\ kJ/mol \qquad (S107)$$

These meaningful energy values (multiplied by the stoichiometric coefficient of 4) are included in Figure S11 below (upper right).

Why $\Delta_r G^o$ matters in an open biological system: Rate-constant ratios. The first law of thermodynamics can be written in very general terms, as $\Delta U = w + q$, only for a process involving a *closed* system, which only energy can enter or leave, while matter cannot. The second law in the form of the inequality of Clausius also applies only to a process involving a closed system, and the same applies to its direct corollary, $\Delta G < w_{ele}$ for a process at constant T and P. However, biological systems are open. Fortunately, ΔG remains relevant even in an open system via its relation to equilibrium and kinetics. For a reaction in a closed system in equilibrium, the standard free energy of reaction, $\Delta_r G^o$, relates to the (unitless) equilibrium constant K according to

$$\Delta_r G^o = -\ RT\ \ln K \qquad (S108)$$

Analysis of the kinetics of an elementary-step reaction in a closed system in equilibrium shows that the equilibrium constant K relates to the rate constants k_f and k_r of forward and reverse reactions, respectively, according to [26]

$$\frac{k_f}{k_r} = \left(\frac{mol}{L}\right)^{\Delta\nu} K \qquad (S109)$$

a well-known relation in enzyme kinetics. The exponent $\Delta\nu$ is the difference between the summed stoichiometric coefficients of products and reactants (excluding H_2O, the solvent). Note that the rate-constant ratio is unaffected by a catalyst, since a catalyst increases both rate

constants proportionally. Combining the thermodynamics and kinetics equations (S108) and (S109) gives

$$\frac{k_f}{k_r} = \left(\frac{mol}{L}\right)^{\Delta v} \exp\left(-\frac{\Delta_r G^o}{RT}\right) \tag{S110}$$

a useful result for biochemistry. The rate constants are essentially microscopic quantities and therefore their ratio does not change significantly when the reaction system is opened up. Thus the ratio of forward and reverse rate constants is determined by $\Delta_r G^o$ even in an open system. For instance, with $\Delta_r G^o$ = -30 kJ/mol at 300 K, $k_f/k_r = 1.7\times10^5$ (mol/L)$^{\Delta v}$ >> 1. This means that the reaction will go forward, unless reactants are scarce and/or products already abundant (qualifications that can be understood both from kinetics and thermodynamics perspectives). If the reaction is due to a series of K elementary reactions, the relation generalizes to [26]

$$\left(\frac{k_{f,1}}{k_{r,1}}\right)\left(\frac{k_{f,2}}{k_{r,2}}\right)\cdots\left(\frac{k_{f,K}}{k_{r,K}}\right) = \left(\frac{mol}{L}\right)^{\Delta v} \exp\left(-\frac{\Delta_r G^o}{RT}\right) \tag{S111}$$

Pure energy transfer: Z- vs. EZ-scheme. The Z-scheme is limited in terms of the processes it can depict cogently. It is well suited for redox reactions, which are shown as arrows connecting two standard-reduction potentials or equivalently half-reaction energy-difference levels; thus, series of redox reactions, or electron transport chains, can be represented quite compactly. It can also show the energy increase due to an absorbed photon, e.g. P680 → P680*, in terms of a vertical arrow. However, reactions outside the redox concept cannot be represented in terms of standard reduction potentials. For instance, ATP + H_2O → ADP + P_i is not a redox reaction and could not be shown in the Z-scheme with its E^o axis, while more general free-energy diagrams can represent this reaction.

Steps in photosynthesis that look disjointed in the Z-scheme include pure energy transfer between corresponding states of P680 and a neighboring chlorophyll. Consider

(1) Photon energy exciting P680:	P680 → P680*
(2) Energy transfer to Chl, forming Chl*:	P680* + Chl → P680 + Chl*
(3) Charge separation boosting Chl* to **Chl**$^{+\cdot}$:	Chl* + Pheo → **Chl**$^{+\cdot}$ + Pheo$^{-\cdot}$
(4) Energy back transfer from **Chl**$^{+\cdot}$ to P680$^{+\cdot}$:	**Chl**$^{+\cdot}$ + P680 → Chl + P680$^{+\cdot}$

This "forth and back" energy transfer between Chl and P680, steps (2) and (4), seems compatible with the "two-step" process discussed in ref.[12].

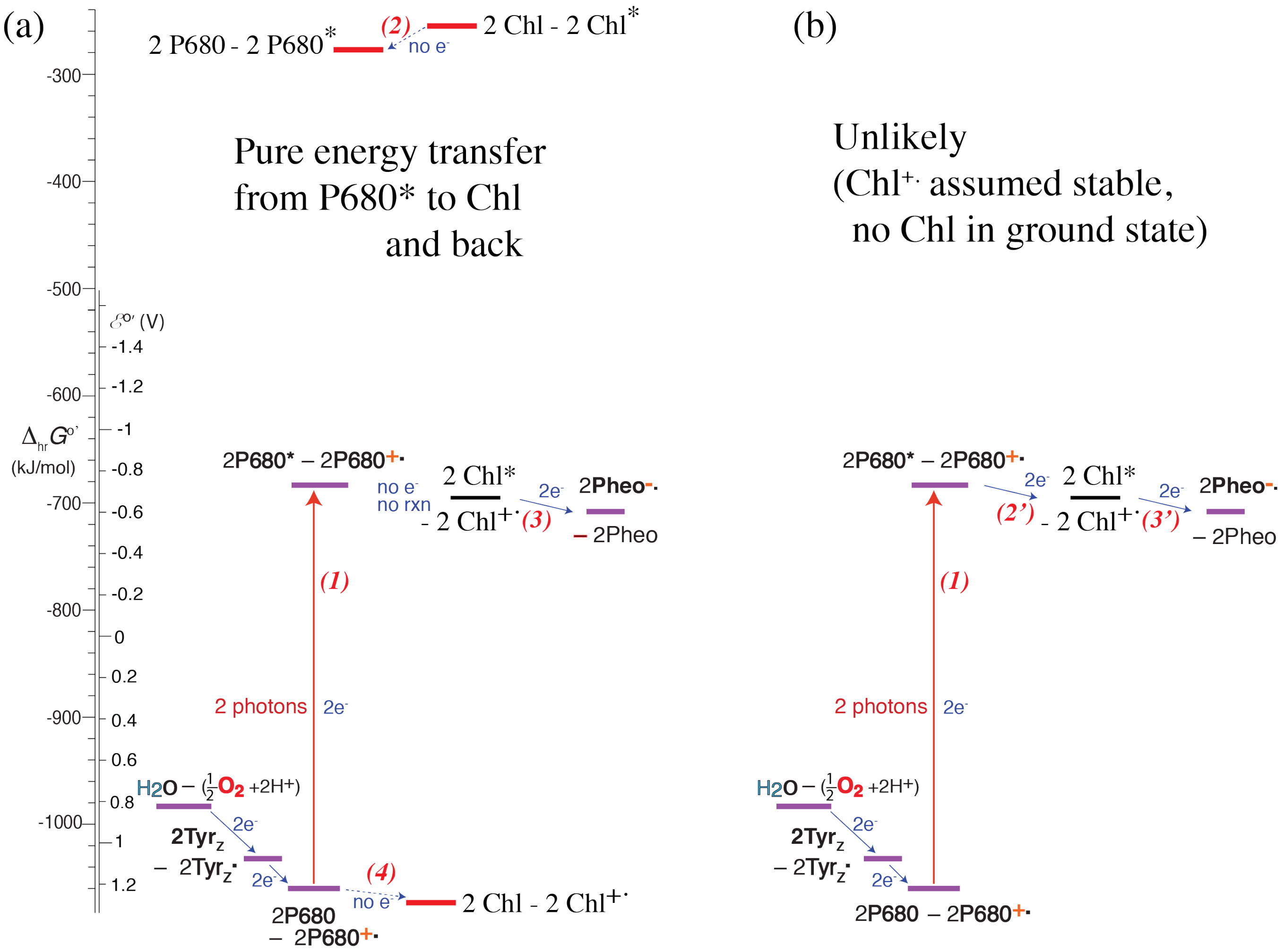

Figure S7. (a) Z-scheme with pure energy transfer from P680* to Chl and back from $Chl^{+\cdot}$ to P680 included. Labels (1) through (4) match the energy-transfer steps specified in the text. The non-redox energy transfer results in unusual disjointed features ("energy levels" labeled in red; as before, G^o has been omitted before each species listed) and a 'break' in the electron transfer chain. (b) Z-scheme with Chl* - $Chl^{+\cdot}$ added into the electron transfer chain, which is unrealistic since it excludes the ground state of Chl and assumes that $Chl^{+\cdot}$ is indefinitely stable. This does not represent the pure energy transfers as in (a).

To represent this chain of events in the Z-scheme one has to introduce non-redox levels, e.g. for Chl* → Chl, see Figure S7a top. In addition, the last process (4) would be an "unexpected" transfer from the second-lowest to the lowest half-reaction energy difference, bottom of Figure S7a. The redox-transfer chain is interrupted (between 2 P680* - 2 $P680^{+\cdot}$ and 2 Chl* - 2 $Chl^{+\cdot}$). Such "disjointed" processes have rarely, if ever, been shown in a Z-scheme.

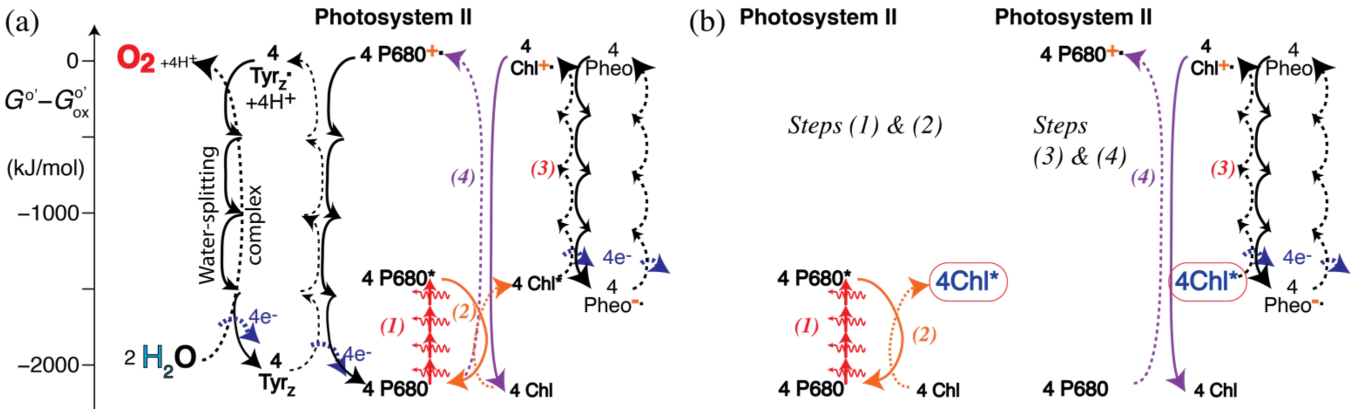

Figure S8. (a) EZ-scheme with pure energy transfer from P680* to Chl and back from $Chl^{+\cdot}$ to P680 included. (b) The energy transfer steps (1) and (2) shown separately for clarify, resulting in Chl*. (c) Steps (3) and (4), i.e., electron transfer from Chl* to pheophytin, generating $Chl^{+\cdot}$, which transfers its energy to P680, converting back to ground state Chl and generating $P680^{+\cdot}$.

One might be tempted to add Chl into the electron transport chain as shown in Figure S7b, but that represents the following, different process:

(1) P680 → P680*

(2’) P680* + **$\underline{Chl^{+\cdot}}$** → $P680^{+\cdot}$ + Chl*

(3’) Chl* + Pheo → **$\underline{Chl^{+\cdot}}$** + $Pheo^{-\cdot}$

It is unclear from where the extremely high-energy reactant **$\underline{Chl^{+\cdot}}$** in step (2’) would come. Also note that the ground state of Chl would never be involved here.

A sequence of RETAR diagrams can represent the sequence of events (1) – (4). Figure S8a shows the full process. Since the back-and-forth energy transfer is somewhat complicated, it is broken up into diagrams showing consecutive events in Figure S8b.

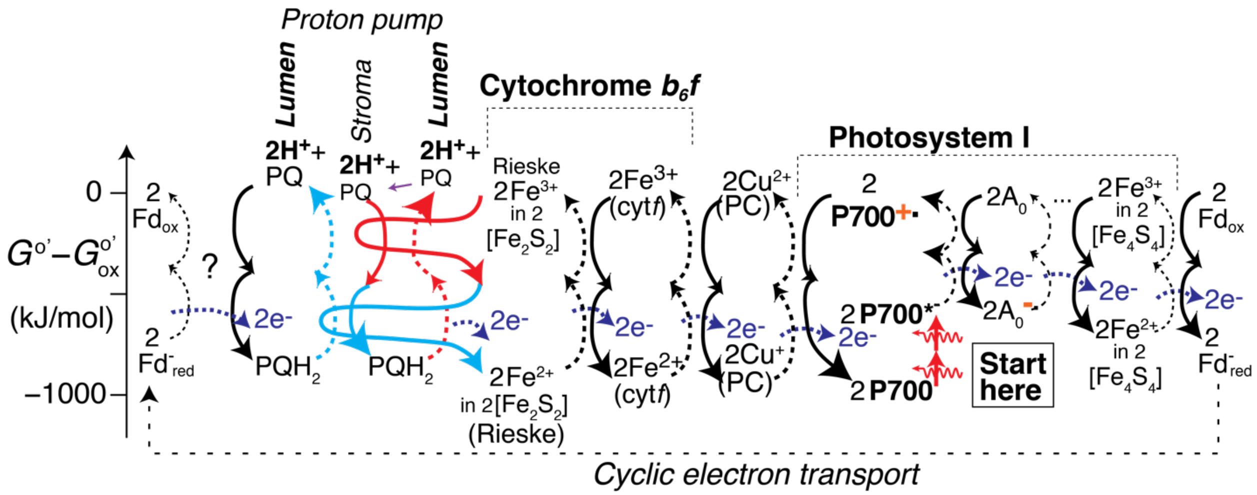

Figure S9. Cyclic electron transport driven by photon energy absorbed in Photosystem I, in the EZ-scheme.

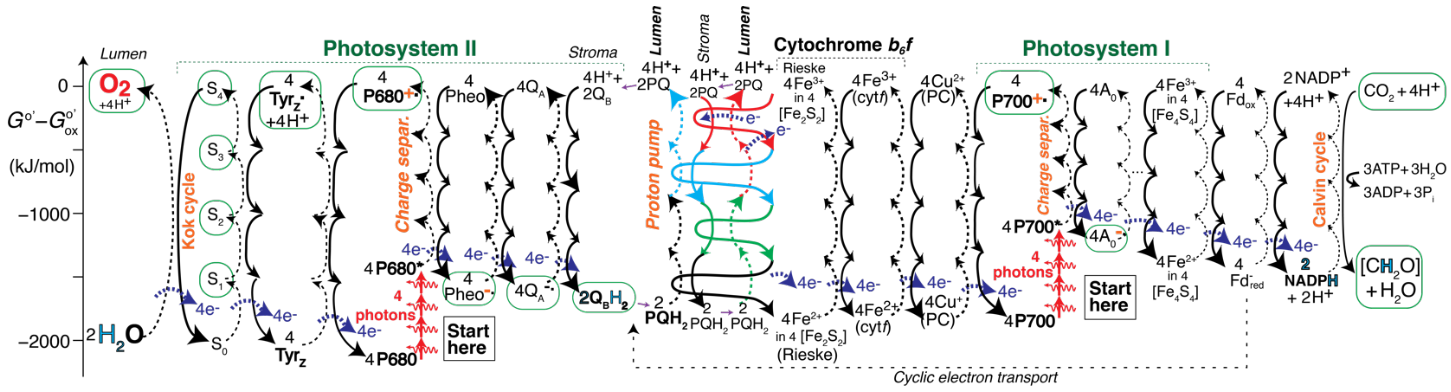

Figure S10. Comprehensive EZ-scheme of photosynthesis in plants. Important species missing from the traditional Z-scheme are marked by green boxes.

Energetics of CO_2 in the Calvin cycle. The EZ-scheme developed in the main text as shown in Figures 3-5 and S10 concludes with the restoration of $NADP^+$ and conversion of CO_2 to carbohydrates. The surprising status of CO_2 among the higher-energy species here is explained and confirmed in the following.

The reduction half reaction in question, simplified relative to the actual Calvin cycle by ignoring phosphorylation, is

$$CO_2 + 4\,H^+ + 4\,e^- \rightarrow [CH_2O] + H_2O$$

$$\Delta_{hr}G^{o'} = -1488\text{ kJ/mol.} \qquad \text{(S112)}$$

The individual free energies at biochemical standard conditions can be found in Table S1 in ref.[8] and divided by the number of pairs of electron-pair bonds:

	CO_2 + 4 H^+		vs.	[CH_2O] + H_2O			
$G^{o'}$ (kJ/mol)	-1529	+680	vs.	-8767/6	-876	kJ/mol,	$\Delta_{hr}G^{o'}$ = -1488 kJ/mol
Per 2 electron-pair bonds:	-765	--		-731	-876	kJ/mol.	(S113)

The data show that in terms of free energy per electron-pair bond, reactants and products are similar, but the reactants have only 4 electron-pair bonds while the products have 6. Bonding by definition reduces the energy or enthalpy, and the entropic effects are minor, so the two additional bonds reduce the free energy of the products by about (-731 – 876)/2 = -804 kJ/mol. The energy of the reduced species is further raised by the ionization energy of hydrogen in water at pH 7, +170 kJ/mol,[8] which can be read off as 340 kJ/mol for 2 H^+(aq) in Figure S4b (center). The total difference between products with (-8767/6 - 876) kJ/mol and reactants with (-1529 + 680) kJ/mol is $\Delta_{hr}G^{o'}$ = -1488 kJ/mol, consistent with the reach of the right-most solid-line downward arrow in Figures 3-5.

CO_2 + 4 H^+ is of high energy compared to the oxidized species in PSI in the sense that it could theoretically react with their reduced counterparts (except ground-state P700) and release energy. An example is

$$CO_2 + 4\,H^+ + 4\,A_0^{-\cdot} \rightarrow [CH_2O] + H_2O + 4\,A_0 \qquad \Delta_{hr}G^{o'} \approx -230 \text{ kJ/mol} \quad \text{(S114)}$$

Note that we *have* to restrict this analysis to reactions with reduced species since CO_2 + 4 H^+, as oxidized species, would not react with oxidized species. CO_2 + 4 H^+ could 'even' spontaneously boost P700* to $P700^{+\cdot}$ and still release energy:

$$CO_2 + 4\,H^+ + 4\,P700^* \rightarrow [CH_2O] + H_2O + 4\,P700^{+\cdot} \quad \Delta_{hr}G^{o'} \approx -350 \text{ kJ/mol} \quad \text{(S115)}$$

Chemical free energy of O_2 vs. glucose. The main result of photosynthesis is the production of glucose and O_2 according to eq.(1). In the main text, the energetics of this reaction were analyzed in terms of the average enthalpy of two electron-pair bonds (in kJ/mol) in the molecules, see eq.(31). Here we repeat the analysis in terms of the average free energy of two electron-pair bonds in the molecules:

$H_2O(l)$	CO_2	$C_6H_{12}O_6$	**O_2**	
-876	-765	-731	**-464** kJ/mol	(S116)

This shows, similarly as with the bond enthalpies, that O_2 has by far the least negative free energy per electron-pair bond. It is clearly the highest-energy species in the reaction.

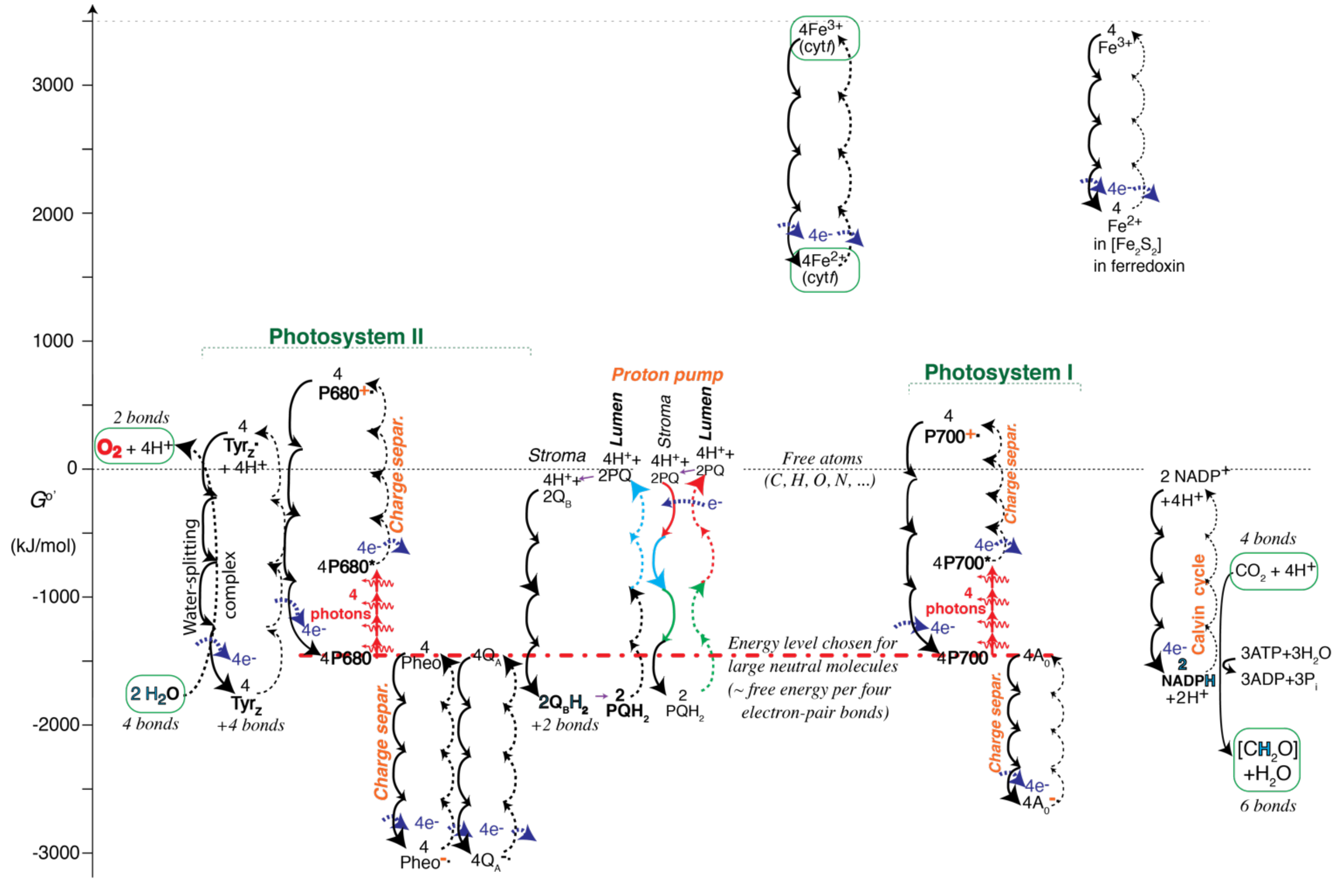

Figure S11. (a) EZ-scheme of photosynthesis with an ‘absolute’ free-energy scale. The individual free energies[8] of small molecules and ions (marked by green boxes) are shown relative to the free atoms and free electrons at zero energy. Where large reduced molecules have additional bonds relative to their oxidized counterparts, the energies of the “active bonds” only are considered. Other larger neutral molecules are assigned, somewhat arbitrarily, an energy slightly above 2 H_2O (dash-dotted horizontal red line), corresponding to their energy in a diagram of free energy per four electron pair bonds.[8, 22] This diagram shows the lower energy of $P700^{+\bullet}$ and of $Tyr_Z^{\bullet}$ compared to $P680^{+\bullet}$ more clearly than the otherwise smoother EZ-scheme in Figures 4 and S10, where all oxidized species are kept at the same level of energy. Of the Fe- and Cu-based redox species, cytochrome *f* and ferredoxin are shown here since their standard free energies of formation are readily available [25] and can be converted as shown in eqs.(S99) – (S107).

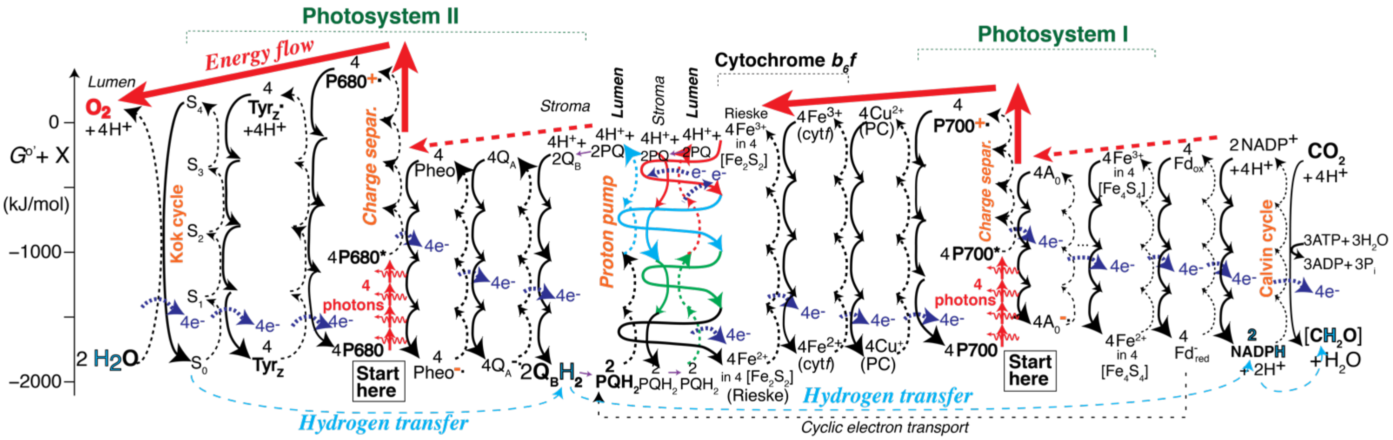

Figure S12. Alternative comprehensive EZ-scheme of photosynthesis in plants, converting H_2O and CO_2 (lower left and upper right corners, respectively) to O_2 and carbohydrate $[CH_2O]$. The vertical shift X of each half-reaction "column" was adjusted to facilitate energetics comparisons, e.g., between $P680^{+\bullet}$ and $P700^{+\bullet}$. Energy flow and net hydrogen transfer are indicated. A simplified version of this scheme is shown in Figure 5.

References Cited

1. Nelson, D.L., and M.M. Cox. *Lehninger Principles of Biochemistry*. 6th ed. New York: Worth Publishers, 2013.
2. Berg, J. M., J. L. Tymoczko, and L. Stryer. *Biochemistry*. 7th ed. New York: W. H. Freeman, 2012.
3. Voet, D., and J. G. Voet. *Biochemistry*. 3rd ed. Hoboken, New Jersey: Wiley, 2004.
4. Karp, G. *Cell and Molecular Biology*. 7th ed. Hoboken, NJ: Wiley, 2013.
5. Morris, J., D. Hartl, A. Knoll, R. Lue, M. Michael, A. Berry, A. Biewener, B. Farrell, N. M. Holbrook, J. Heitz, M. Hens, J. Merrill, R. Phillis, D. Pires, and E. Lozovsky. *Biology: How Life Works*. 3rd ed: W. H. Freeman, 2019.
6. Govindjee, D. Shevela, and L. O. Björn. "Evolution of the Z-scheme of photosynthesis: a perspective." *Photosynth. Res.* 133 (2017): 5-15.
7. Dill, K. A., and S. Bromberg. *Molecular Driving Forces*. 2nd ed. London/New York: Garland Science, 2011.
8. Schmidt-Rohr, K. "Oxygen is the High-Energy Molecule Powering Complex Multicellular Life: Fundamental Corrections to Traditional Bioenergetics." *ACS Omega* 5 (2020): 2221-33.

9. Blankenship, R. E. *Molecular Mechanisms of Photosynthesis*. 2nd ed. Chichester: Wiley Blackwell, 2014.
10. Johnson, M. P. "Photosynthesis." *Essays Biochem.* 60 (2016): 255-73.
11. McConnell, I., G. Li, and G. W. Brudvig. "Energy Conversion in Natural and Artificial Photosynthesis." *Chem. Bio. Review* 17 (2010): 434-46.
12. Caffari, S., T. Tibiletti, R. C. Jennings, and S. Santabarbara. "A Comparison Between Plant Photosystem I and Photosystem II Architecture and Functioning." *Curr. Prot. Pept. Sci.* 15 (2014): 296-331.
13. Mungan, C. E. "Radiation thermodynamics with applications to lasing and fluorescent cooling." *Am. J. Phys.* 73 (2005): 315-22.
14. Spanner, D. C. *Introduction to Thermodynamics*. New York: Academic Press, 1964.
15. Kondepudi, D., and I. Prigogine. *Modern Thermodynamics*. 2nd ed. Chichester: John Wiley & Sons, 2015.
16. Delgado-Bonal, A. "Entropy of radiation: the unseen side of light." *Sci. Reports* 7 (2017): 1642: 1-11.
17. Yourgrau, W., and A. van der Merwe. "Entropy Balance in Photosynthesis." *Proc. Nat. Acad. Sci. U.S.A.* 59 (1968): 734-37.
18. Planck, M. *The Theory of Heat Radiation*. New York: Dover Publications, 1959.
19. Baierlein, R. *Thermal Physics*: Cambridge University Press, 1999.
20. Knox, R. S. "Thermodynamics and the Primary Processes of Photosynthesis." *Biophys. J.* 9 (1969): 1351-62.
21. Albarran-Zavala, E., and F. Angulo-Brown. "A Simple Thermodynamic Analysis of Photosynthesis." *Entropy* 9 (2007): 152-68.
22. Schmidt-Rohr, K. "Why Combustions Are Always Exothermic, Yielding About 418 kJ per Mole of O_2." *J. Chem. Educ.* 92 (2015): 2094-99.
23. Schmidt-Rohr, K."How Batteries Store and Release Energy: Explaining Basic Electrochemistry." *J. Chem. Educ.* 95 (2018): 1801-10.
24. Bockris, J. O. , A. K N. Reddy, and M. Gamboa-Aldeco. *Modern Electrochemistry 2A, Fundamentals of Electrodics*. 2nd ed. New York: Kluwer Academic / Plenum, 2001.
25. Alberty, R. A. *Thermodynamics of Biochemical Reactions*. Hoboken, NJ: Wiley, 2003.
26. Oxtoby, D. W., H. P. Gillis, and L. J. Butler. *Principles of Modern Chemistry*. 8th ed. Boston, MA: CENGAGE Learning, 2015.
